# Multimodal network alterations in diagnosis-age-defined early- and late-onset Alzheimer’s disease

**DOI:** 10.3389/fnins.2026.1914358

**Published:** 2026-09-08

**Authors:** Zesong Yuan, Jing Wang, Baohua Cheng, Yingpeng Kuang, Rongyu Zhang, Nana Luo, Shijun Qiu, Yujie Liu

**Affiliations:** 1 First Clinical Medical College of Guangzhou University of Chinese Medicine, Guangzhou, China; 2 Department of Radiology, The First Affiliated Hospital of Guangzhou University of Chinese Medicine, Guangzhou, China; 3 State Key Laboratory of Traditional Chinese Medicine Syndrome, Guangzhou, China

**Keywords:** Alzheimer’s disease, early-onset Alzheimer’s disease, late-onset Alzheimer’s disease, multimodal MRI, structural-functional coupling

## Abstract

**Introduction:**

Early-onset Alzheimer’s disease (EOAD) and late-onset Alzheimer’s disease (LOAD) may differ in large-scale network organization. We compared multimodal MRI markers of diagnosis-age-defined Alzheimer’s phenotypes.

**Methods:**

Alzheimer’s Disease Neuroimaging Initiative participants were classified by age at first recorded mild cognitive impairment or Alzheimer’s disease diagnosis: <65 years for EOAD and ≥65 years for LOAD. Retrospective symptom-onset information was consistent with this grouping when available. We assessed resting-state functional measures, graph-theoretical nodal metrics, structural-functional coupling (SFC), structural decoupling, and DTI-derived measures. Primary models adjusted for age and sex; sensitivity analyses examined nonlinear age, covariate overlap, clinical severity, disease stage, atrophy, biomarkers, head motion, regional coverage, and control-group composition.

**Results:**

The functional cohort included 50 EOAD, 72 LOAD, and 113 healthy controls. Global mean SFC showed only a modest group effect. LOAD showed higher regional SFC than EOAD in four frontal regions after false discovery rate correction. Graph-theoretical effects were predominantly nominal: only the overall three-group effect in Vermis 9 nodal efficiency survived within-family correction, and no EOAD-versus-LOAD nodal contrast survived. DTI-derived regional measures and structural decoupling were less subtype-discriminative. All four frontal effects remained significant after mean Power framewise displacement adjustment and overlap weighting, with bootstrap confidence intervals excluding zero. However, corrected support narrowed to one region in the nonlinear-age model and disappeared in disease- stage-, APOE ε 4-, amyloid PET-, and centiloid-adjusted models; restriction to amyloid-negative controls also retained one region.

**Conclusion:**

In the primary model, diagnosis-age-defined LOAD showed higher frontal regional SFC than EOAD. The absence of corrected EOAD-versus-LOAD graph findings, variable sensitivity-analysis support, and lack of corrected SFC–clinical associations indicate a model-sensitive network phenotype rather than a definitive onset-age biomarker or clinically validated subtype marker.

## Highlights

- LOAD showed higher frontal SFC than diagnosis-age-defined EOAD.
- Frontal SFC effects persisted after motion adjustment and overlap weighting.
- SFC was more subtype-sensitive than graph, SDI, and regional DTI measures.

## 1 Introduction

Alzheimer’s disease is biologically and clinically heterogeneous, and age at onset is one of the most consistent sources of that heterogeneity (Scheltens et al., 2021;Lam et al., 2013). Earlier-onset disease is often associated with broader neocortical involvement, faster systems-level failure, and relatively prominent non-amnestic features, whereas later-onset disease more often presents with a memory-predominant phenotype (Valdez-Gaxiola et al., 2024; Seath et al., 2024). Even so, the imaging features that most reliably distinguish early-onset Alzheimer’s disease (EOAD) from late-onset Alzheimer’s disease (LOAD) remain uncertain, particularly when multiple imaging domains are considered within the same analytic framework (Valdez-Gaxiola et al., 2024; Seath et al., 2024; Heo et al., 2024; Ottoy et al., 2025). This uncertainty matters because EOAD and LOAD are clinically useful subgroups, yet it remains unclear whether they are best separated by regional disease burden, altered network organization, or a combination of both.

A key reason for this uncertainty is that EOAD–LOAD differences may not be captured fully by isolated regional abnormalities. Regional atrophy, hypometabolism, and focal functional alterations are important markers of Alzheimer-related injury, but age-related heterogeneity may also be expressed in how brain damage is distributed and coordinated across large-scale systems (Valdez-Gaxiola et al., 2024; Seath et al., 2024; Heo et al., 2024). A network-oriented analysis is therefore useful because it can test whether EOAD and LOAD differ mainly in focal tissue burden or in the way structural backbones and functional dynamics are organized across the connectome (Heo et al., 2024; Ottoy et al., 2025). This provides the rationale for moving from isolated regional markers toward coordinated measures of brain network architecture.

Most comparative imaging studies have emphasized regional atrophy, metabolism, or conventional functional connectivity (Ottoy et al., 2025). These approaches are informative, but they do not fully test whether subtype-related heterogeneity is expressed through the alignment between structural connectivity and functional communication, or through changes in nodal topology within large-scale networks (Heo et al., 2024; Ottoy et al., 2025). A multimodal network framework can address this question more directly by jointly examining structure-function alignment, graph-theoretical topology, local functional activity, and DTI-derived regional measures (Ottoy et al., 2025; Sun et al., 2024; Lu et al., 2025; Rubinov and Sporns, 2010). Such a design allows the analysis to ask not only where abnormalities are present, but also which analytic level provides the clearest subtype-sensitive signal.

Structural-functional coupling is particularly relevant because it quantifies how closely functional interactions follow the underlying structural connectivity scaffold (Sun et al., 2024; Lu et al., 2025). Graph-theoretical nodal measures provide complementary information on integration, segregation, and hubness within the brain network (Rubinov and Sporns, 2010). Local functional indices and DTI-derived regional metrics can further place the main network findings in a broader multimodal context. We therefore used multimodal Alzheimer’s Disease Neuroimaging Initiative data to compare EOAD, LOAD, and healthy controls within a unified framework (Petersen et al., 2010; Jack et al., 2008). We hypothesized that subtype differences would be most evident at the systems-network level and that frontal structure-function coupling, supported by graph-theoretical topology, would provide informative separation between EOAD and LOAD.

## 2 Materials and methods

### 2.1 Data source and participants

Data used in the preparation of this article were obtained from the Alzheimer’s Disease Neuroimaging Initiative (ADNI) database.^1^ ADNI was launched in 2003 as a public-private partnership, led by Principal Investigator Michael W. Weiner, MD. The primary goal of ADNI has been to test whether serial magnetic resonance imaging, positron emission tomography, other biological markers, and clinical and neuropsychological assessments can be combined to measure the progression of mild cognitive impairment and early Alzheimer’s disease. For up-to-date information, see footnote 1.

Participants on the Alzheimer continuum were operationally classified as diagnosis-age-defined EOAD or LOAD according to whether the first recorded diagnosis of mild cognitive impairment or Alzheimer’s disease occurred before 65 years of age or at 65 years and older. Healthy controls were drawn from cognitively normal ADNI participants. The functional MRI cohort included 50 participants with EOAD, 72 with LOAD, and 113 healthy controls. Analyses requiring DTI-derived structural measures, structural connectivity, or structural-functional coupling were restricted to participants with usable DTI and functional data after quality control, yielding a matched multimodal subset of 47 participants with EOAD, 69 with LOAD, and 111 healthy controls. Participants were excluded from a given modality-specific analysis when the required imaging dataset was unavailable or failed quality control, so analytic sample size varied across modalities according to data availability and usability. To verify the onset-age consistency of the EOAD/LOAD assignment, estimated cognitive symptom-onset age was derived, when available, from ADNI participant demographic records and participant-reported cognitive symptom onset records (PTDEMOG and PTCOGBEG datasets). Five participants in the EOAD group and two participants in the LOAD group lacked sufficient symptom-onset information in the PTCOGBEG dataset. Among all remaining Alzheimer-spectrum participants, the estimated cognitive symptom-onset age was consistent with the assigned EOAD/LOAD group, and no cross-group reassignment was required. Analytic cohort flow and modality-specific sample coverage are summarized in Supplementary Figure 1.

### 2.2 Clinical assessment

Clinical and demographic variables included age, sex, education years, Functional Activities Questionnaire (FAQ) total score, Geriatric Depression Scale (GDS) total score, Mini-Mental State Examination (MMSE), Montreal Cognitive Assessment (MoCA), Clinical Dementia Rating-Sum of Boxes (CDR-SB), ADAS cognitive total score, disease stage, APOE epsilon4 carrier status, amyloid PET status, and amyloid centiloids when available. Disease stage was summarized from ADNI diagnostic records as cognitively normal, mild cognitive impairment, or dementia. APOE epsilon4 carrier status was derived from APOE genotype data, and amyloid biomarker status was summarized using amyloid PET status and centiloids when available.

Age and sex were retained as core covariates in the primary imaging models. Education, cognitive/functional severity measures, affective symptom burden, disease stage, APOE epsilon4 carrier status, and amyloid PET measures were included in additional models when relevant and available. Expanded models used available-case analyses for the added covariates to avoid introducing artificial information through imputation. Missingness was therefore reported descriptively, and model-specific sample sizes varied according to covariate availability and ROI-level coupling coverage.

### 2.3 MRI acquisition and ComBat harmonization

Magnetic resonance imaging data were obtained from the Alzheimer’s Disease Neuroimaging Initiative (ADNI) across multiple study phases and acquisition sites (Jack et al., 2008). As a multicenter longitudinal study, ADNI implemented standardized imaging protocols and centralized quality control procedures, although scanner vendors, hardware platforms, and sequence parameters varied across phases and sites (Jack et al., 2008).

Resting-state functional MRI and diffusion tensor imaging data were acquired within the heterogeneous multicenter ADNI framework (Jack et al., 2008). To reduce non-biological variability, ComBat harmonization was applied to preprocessed images rather than to derived connectivity or summary measures (Johnson et al., 2007). Functional images were harmonized after spatial normalization, nuisance regression, and temporal band-pass filtering; resting-state functional connectivity, ALFF, fALFF, and ReHo were then calculated from the harmonized functional images and were not harmonized a second time. Preprocessed DTI NIfTI images were harmonized independently, before DTI regional measures, tractography-based structural connectivity, structural-functional coupling, and structural decoupling were derived. ADNI phase, scanner manufacturer, and scanning protocol were entered as batch-related variables, whereas diagnostic group, age, and sex were included as biological covariates intended to preserve clinically relevant variation. Diagnostic group was therefore not treated as a batch effect. Functional and DTI pipelines were harmonized separately, and no pooling of derived metrics across modalities was performed.

### 2.4 Resting-state functional MRI processing

Resting-state fMRI preprocessing was performed using DPABI version 9.0 and DPARSF version 5.5 (Yan and Zang, 2010; Yan et al., 2016) within the SPM framework (Ashburner and Friston, 2005; Ashburner, 2007; Tierney et al., 2025). After discarding the first 10 volumes, images underwent slice-timing correction, rigid-body realignment, coregistration to the T1-weighted image, tissue segmentation, and DARTEL normalization to Montreal Neurological Institute space at 3-mm isotropic resolution. Nuisance regression included a first-order polynomial trend, Friston-24 head-motion parameters (Friston et al., 1996), white-matter and cerebrospinal-fluid nuisance signals derived from SPM prior masks thresholded at 0.99, and motion-scrubbing regressors. Volumes with Power framewise displacement greater than 0.5 mm, together with the preceding two volumes and the following one volume, were identified as motion-contaminated and modeled as separate nuisance regressors (Power et al., 2012). Global-signal regression was not applied. Participants with translation greater than 3 mm were excluded. Temporal filtering was performed at 0.01–0.10 Hz. Mean Power framewise displacement was also used for group-level motion comparisons and as an additional covariate in sensitivity models. Although spatially smoothed derivative images were generated during the broader preprocessing workflow, the smoothed outputs were not used for ROI-based functional connectivity, seed-based functional connectivity, or graph-theoretical analysis. ALFF, fALFF, and ReHo were computed using the standard DPABI/DPARSF implementations (Yan and Zang, 2010; Yan et al., 2016; Zang et al., 2004, 2007; Zou et al., 2008).

For ROI-based network construction, the harmonized, unsmoothed FunImgARCWF images were used. The mean time series was extracted from each of the 116 AAL regions, and pairwise Pearson correlations between regional time series were Fisher z-transformed to generate a symmetric 116 × 116 functional-connectivity matrix for each participant. The diagonal was excluded from subsequent analyses, and the AAL label order was kept identical across functional and structural matrices. No additional nuisance regression, spatial smoothing, or global-signal manipulation was applied during subsequent ROI time-series extraction or functional-connectivity matrix construction.

Exploratory seed-based resting-state functional-connectivity analyses used atlas-defined seed masks specified from the AAL116 template with the DPARSF Define ROI function (Tzourio-Mazoyer et al., 2002). For each atlas-defined seed, the mean seed time series was extracted from the harmonized, unsmoothed FunImgARCWF images and correlated with the time series of each voxel within the analysis mask. Correlation coefficients were Fisher z-transformed before group-level analysis. The resulting seed-to-voxel maps were entered into general linear models adjusted for age and sex, and voxel-level FDR correction was applied to the reported group contrasts. No additional spatial smoothing or global-signal regression was applied during seed-based map construction.

### 2.5 Diffusion tensor imaging processing and structural connectivity reconstruction

Diffusion tensor imaging data were converted from DICOM to NIfTI with dcm2niix and were visually inspected before Linux-based processing with FSL version 6.0.7.16 and FreeSurfer version 7.4.1. For each participant, the first b = 0 volume was extracted with fslroi, brain extraction was performed with BET using a fractional intensity threshold of 0.30, and eddy-current and head-motion correction was performed with eddy_correct using the first volume as the reference. DTI-derived maps, including fractional anisotropy, mean diffusivity, and eigenvalue-derived measures, were estimated with dtifit from the corrected DTI series, brain mask, b-values, and b vectors. High-resolution T1-weighted images were processed with FreeSurfer recon-all -all for subject-specific anatomical reconstruction (Fischl, 2012), and voxelwise fiber-orientation distributions were estimated with bedpostx. Datasets with incompatible DTI volumes and b-value/b-vector files, missing corrected DTI images or masks, incomplete bedpostx output, or failed quality control were excluded before multimodal subject matching.

The AAL116 atlas was mapped from MNI space to each participant’s DTI space through an explicit linear transform chain. T1-to-DTI registration used a six-degree-of-freedom FLIRT transformation to the brain-extracted b = 0 image. T1-to-MNI152 registration used a 12-degree-of-freedom FLIRT transformation, which was inverted to obtain MNI-to-T1 and concatenated with T1-to-DTI to obtain the MNI-to-DTI transform. The AAL atlas was resampled with nearest-neighbor interpolation and separated into 116 binary subject-specific ROI masks (Tzourio-Mazoyer et al., 2002). Registration alignment, ROI coverage, nonempty masks, and DTI-image usability were reviewed during quality control.

Probabilistic tractography was run separately from each AAL ROI with probtrackx2, using all 116 ROI masks as targets, the bedpostx fiber samples, and the participant’s b = 0 brain mask. The final batch configuration used 2,000 samples per seed voxel, a maximum of 2,000 steps, a 0.5-mm step length, curvature threshold 0.20, fiber threshold 0.01, loop checking, and one-way target conditioning. For each seed ROI i, the tractography distribution in seeds_to_targets.nii.gz was summarized within each target ROI j. Directed connection strength SC(i,j) was obtained from the target-mask tractography signal, the diagonal was set to zero, target-size normalization was implemented using the mean signal within the target ROI, and reciprocal directions were averaged to form a symmetric weighted 116 × 116 matrix. Structural and functional files were matched by group and participant identifier, and only participants with both usable matrices entered coupling and structural-decoupling analyses.

### 2.6 Structural-functional coupling

Structural-functional coupling was evaluated in the matched multimodal cohort (Sun et al., 2024; Lu et al., 2025). The target-size-normalized, symmetrized structural matrix and Fisher-z functional matrix were aligned by the identical AAL116 row and column order. For each region, structural weights linking that node to the remaining nodes were paired with the corresponding functional-connectivity values. The diagonal was excluded; zero structural edges and non-finite structural or functional values were omitted, but negative functional-connectivity values were retained. No additional absolute-value transformation or proportional threshold was applied to functional connectivity. Nonzero structural weights were transformed with log1p, and regional coupling was defined as the Spearman correlation between transformed structural weights and corresponding functional-connectivity values. At least three valid edges were required for a regional estimate. Subject-level mean coupling was calculated as the average of all finite regional coupling values.

Because tractography coverage and the number of nonzero structural edges varied across participants and regions, the number of contributing edges and the proportion of participants with calculable regional coupling were quantified. The four principal frontal ROIs were additionally examined with valid-edge count models and group-stratified bootstrap analyses. Interpretation therefore emphasized FDR-corrected regional estimates together with ROI-specific calculability rather than relying on the global mean alone.

### 2.7 Structural decoupling index

Structural decoupling index was computed from the subject-specific structural-connectivity matrix and the corresponding AAL116 ROI time series using graph-frequency decomposition following Preti and Van De Ville (2019). Before decomposition, the structural matrix was symmetrized, its diagonal was set to zero, and nonfinite or negative weights were set to zero. A degree-normalized graph Laplacian was then constructed and eigen decomposed to obtain structural harmonics ordered by ascending eigenvalue from low to high graph frequency. The ROI time series were temporally mean-centered and projected onto the resulting structural eigenbasis. Because the parcellation contained 116 nodes, the first 58 eigenmodes were assigned to the low-graph-frequency component and the remaining 58 eigenmodes to the high-graph-frequency component. These components were separately reconstructed in the node domain. For each ROI, the magnitude of each reconstructed component was quantified as its Euclidean norm across time. Regional SDI was calculated as the base-2 logarithm of the ratio of the high-graph-frequency magnitude to the low-graph-frequency magnitude, with a small numerical constant added to both terms to avoid division by zero. Larger SDI values indicate a greater relative contribution of high-graph-frequency functional activity and therefore greater functional deviation from the low-frequency structural scaffold. Analyses used the same matched structural-connectivity and fMRI sample as the coupling analysis and retained only finite regional estimates.

### 2.8 Graph-theoretical analysis

Graph-theoretical analyses were performed using GRETNA version 2.0 (Wang et al., 2015). Subject-specific Fisher-z-transformed AAL116 functional-connectivity matrices generated by DPARSF were imported as network inputs. Only positive functional connections were retained. At each network sparsity threshold, the strongest positive connections were retained and binarized, thereby equating network density across participants. Negative functional connections were excluded from the graph-theoretical analysis, whereas negative functional-connectivity values were retained in the regional structural-functional coupling calculation. Binary networks were evaluated across sparsity thresholds from 0.08 to 0.50 in increments of 0.01. The lower bound approximated the criterion that average nodal degree should exceed 2log(N) for the AAL116 parcellation. Nodal betweenness centrality, degree centrality, nodal efficiency, nodal local efficiency, nodal clustering coefficient, and nodal shortest path length were calculated at each threshold and summarized by area under the sparsity curve to reduce dependence on any single threshold (Rubinov and Sporns, 2010). Network visualizations were generated using BrainNet Viewer (Xia et al., 2013). Group comparisons of nodal area-under-the-curve values were performed using general linear models adjusted for age and sex. Direct EOAD-versus-LOAD two-group tests were distinguished from the three-group omnibus and post hoc workflows. For each analysis framework, graph metric, and contrast, Benjamini-Hochberg FDR correction was applied separately across all 116 AAL nodes. Raw *P*-values and FDR *q*-values were retained; Supplementary Table 3 reports all nodes with nominal *P* < 0.05 together with their corresponding within-family *q*-values.

### 2.9 Statistical analysis

Clinical and demographic variables were summarized using means and standard deviations or counts and percentages, as appropriate. Overall three-group comparisons and the primary EOAD-versus-LOAD contrast were reported separately. MCI and dementia participants were pooled within their respective diagnosis-age-defined EOAD or LOAD groups in the primary analyses; an AD-only sensitivity model additionally adjusted for MCI-versus-dementia stage. Mean structural-functional coupling was evaluated using a general linear model adjusted for age and sex. Whole-brain regional analyses assessed group differences in structural-functional coupling, structural decoupling index, and DTI-derived measures across atlas-defined regions. For each regional feature family and contrast, Benjamini-Hochberg FDR correction was applied across the 116 AAL regions (Benjamini and Hochberg, 1995). Graph-theoretical nodal analyses used the within-family Benjamini-Hochberg procedure described above.

The analysis hierarchy was specified before interpretation of the results. ROI-level structural-functional coupling was the primary subtype-sensitive imaging domain, and nodal graph-theoretical measures were treated as a secondary network-level domain. Local functional metrics, seed-based resting-state functional connectivity, structural decoupling index, and DTI-derived regional measures were exploratory or contextual domains and retained their prespecified domain-specific correction procedures; they were not pooled with the primary SFC correction family. Clinical associations were evaluated for five structural-functional coupling markers—global mean SFC and the four principal frontal regional SFC measures—against six clinical scales, yielding 30 tests. The 30 raw *P*-values were jointly corrected using the Benjamini-Hochberg procedure; graph-theoretical, ALFF, and fALFF markers were not included in this clinical-association family.

Sensitivity analyses repeated the primary models after excluding group-specific imaging outliers more than three standard deviations from the group mean. Alternative age handling included a quadratic-age model (group + age + age squared + sex + education), group-by-age interactions, and a disease-only overlap-weighted LOAD-versus-EOAD analysis with weights estimated from age, sex, and education. Additional available-case models separately included education; MMSE, MoCA, FAQ, GDS, CDR-SB, or ADAS; MCI-versus-dementia stage; APOE ε4 carrier status; amyloid PET positivity; or centiloid values. Atrophy-adjusted models included bilateral hippocampal volume normalized by estimated total intracranial volume, and separate interaction models tested group-by-sex effects. Clinical associations were estimated in the combined EOAD and LOAD sample with adjustment for age, sex, and subgroup. All primary analyses used two-sided inference with the stated multiple-comparison correction.

Additional sensitivity analyses assessed head motion, regional SFC calculability, valid-edge coverage, bootstrap stability, and control-group amyloid composition. Mean Power framewise displacement was compared across groups after adjustment for age and sex and was then added to the three-group ROI-level coupling models. For the four frontal ROIs, calculability and valid-edge counts were summarized by group; valid-edge count models adjusted for age and sex, with additional models incorporating ADNI phase or MCI-versus-dementia stage. LOAD-versus-EOAD effects were evaluated using 5,000 bootstrap repetitions stratified within EOAD and LOAD among participants with calculable regional coupling. Finally, the three-group coupling analysis was repeated after restricting healthy controls to amyloid-negative participants. Model-specific complete-case sample sizes were determined by the availability of the outcome, imaging marker, and covariates; no missing values were imputed.

The ADNI study was approved by the Institutional Review Boards of all participating institutions, and written informed consent was obtained from all participants or their authorized representatives. Because the present study was based exclusively on de-identified, publicly available data and involved no direct participant contact or intervention, it was exempt from further ethical review by our institutional review board.

## 3 Results

### 3.1 Study sample, clinical characteristics, and analytic coverage

A total of 50 EOAD, 72 LOAD, and 113 healthy controls were included in the functional cohort. Analyses requiring DTI-derived structural measures, structural connectivity, or structural-functional coupling used the matched multimodal subset with usable DTI and functional data after quality control (47 EOAD, 69 LOAD, and 111 healthy controls). Estimated cognitive symptom-onset age was available for all but five EOAD and two LOAD participants; all available estimates were consistent with the assigned diagnosis-age-defined group (Table 1). The four principal frontal ROI models included 195, 170, 177, and 176 complete cases, respectively, reflecting ROI-specific SFC calculability (Table 2 and Supplementary Table 1). The transition from the functional cohort to the matched multimodal cohort and the four ROI-specific regional SFC samples is shown in Supplementary Figure 1.

**TABLE 1:** Demographic, clinical, disease-stage, APOE, and amyloid PET characteristics.

Variable	EOAD (*n* = 50)	LOAD (*n* = 72)	HC (*n* = 113)	Overall *p*-value	EOAD vs. LOAD *p*-value
Age, years	60.82 ± 2.49	76.04 ± 7.60	70.12 ± 8.34	<0.001	<0.001
Female, *n* (%)	36/50 (72.0%)	29/72 (40.3%)	72/113 (63.7%)	<0.001	<0.001
Education, years	15.82 ± 2.46	16.54 ± 2.36	16.58 ± 2.22	0.134	0.108
MMSE	27.34 ± 2.60	27.08 ± 2.64	29.18 ± 1.03	<0.001	0.595
MoCA	21.93 ± 4.20	21.07 ± 3.29	26.12 ± 2.32	<0.001	0.229
FAQ	4.14 ± 5.09	3.95 ± 5.35	0.07 ± 0.41	<0.001	0.841
GDS	2.38 ± 1.99	1.89 ± 1.62	0.88 ± 1.10	<0.001	0.152
CDR-SB	1.80 ± 1.46	1.57 ± 1.33	0.05 ± 0.16	<0.001	0.376
ADAS TOTSCORE	10.52 ± 6.30	11.58 ± 7.36	5.15 ± 2.49	<0.001	0.396
Disease stage: MCI, *n* (%)	43/50 (86.0%)	62/72 (86.1%)	–	–	1.000
Disease stage: Dementia, *n* (%)	7/50 (14.0%)	10/72 (13.9%)	–	–	1.000
APOE ε4 carrier, *n*/available (%)	17/43 (39.5%)	28/63 (44.4%)	37/95 (38.9%)	0.775	0.691
Amyloid PET positive, n/available (%)	15/41 (36.6%)	34/61 (55.7%)	27/96 (28.1%)	0.002	0.070
Centiloids	34.37 ± 51.19	43.57 ± 47.49	15.34 ± 24.87	<0.001	0.362

Values are shown as mean ± SD or *n*/available (%), unless otherwise indicated. Both the overall three-group *P*-values and the EOAD-versus-LOAD *P*-values are unadjusted descriptive tests. Sex is shown as female *n* (%). Healthy controls were clinically defined as cognitively normal participants and were not restricted to amyloid-negative individuals; therefore, APOE ε4 carrier status, amyloid PET positivity, and centiloid values are also reported for the control group when available. Percentages for APOE ε4 carrier status and amyloid PET positivity were calculated using participants with available data. ADAS, Alzheimer’s Disease Assessment Scale; APOE, apolipoprotein E; CDR-SB, Clinical Dementia Rating-Sum of Boxes; EOAD, early-onset Alzheimer’s disease; FAQ, Functional Activities Questionnaire; GDS, Geriatric Depression Scale; HC, healthy control; LOAD, late-onset Alzheimer’s disease; MCI, mild cognitive impairment; MMSE, Mini-Mental State Examination; MoCA, Montreal Cognitive Assessment; PET, positron emission tomography; SD, standard deviation.

**TABLE 2:** Frontal regional structural-functional coupling differences in the late-onset Alzheimer’s disease (LOAD) vs. early-onset Alzheimer’s disease (EOAD) comparison.

Region	*N*	β	95% CI	*P*-value	FDR *q*-value
Right middle frontal gyrus	195	0.257	[0.115, 0.399]	0.0005	0.032
Right orbital middle frontal gyrus	170	0.321	[0.140, 0.501]	0.0007	0.032
Right inferior frontal triangular gyrus	177	0.242	[0.102, 0.383]	0.0009	0.032
Left superior medial frontal gyrus	176	0.219	[0.090, 0.348]	0.0011	0.032

Estimates are from age- and sex-adjusted region of interest (ROI)-level structural-functional coupling models including EOAD, LOAD, and healthy controls. β-values represent the adjusted LOAD-versus-EOAD effect; positive values indicate higher coupling in LOAD. False discovery rate (FDR) *q*-values were corrected across 116 atlas-defined regions using the Benjamini-Hochberg procedure. *N* is the total ROI-specific complete-case sample across all three groups.

As expected from the 65-year grouping boundary, EOAD participants were younger than LOAD participants, and sex distribution also differed; age and sex were therefore included in the core models. EOAD and LOAD did not differ significantly in education, MMSE, MoCA, FAQ, GDS, CDR-SB, ADAS total score, disease stage, APOE ε4 carrier status, amyloid PET positivity, or centiloids in the available data. Overall three-group and EOAD-versus-LOAD *P*-values are reported separately because they address different comparisons.

### 3.2 Mean and regional structural-functional coupling

Mean structural-functional coupling showed only a modest overall group effect after adjustment for age and sex, and no pairwise contrast of the mean measure reached nominal significance. Regionally, LOAD showed higher coupling than EOAD in the right middle frontal gyrus, right orbital middle frontal gyrus, right inferior frontal triangular gyrus, and left superior medial frontal gyrus after FDR correction across 116 regions. No disease-versus-control regional contrast survived FDR correction. After additional adjustment for mean Power framewise displacement, all four LOAD-versus-EOAD effects remained FDR-significant (all *q* = 0.031; Table 2 and Supplementary Table 2).

Across the four frontal ROIs, regional SFC was calculable in 69.4%–88.4% of participants, depending on ROI and group. Valid-edge counts did not differ between LOAD and EOAD after adjustment for age and sex, after additional adjustment for ADNI phase, or after disease-stage adjustment. In 5,000 group-stratified bootstrap repetitions, all four confidence intervals excluded zero and 98.18%–99.94% of estimates retained the positive LOAD-versus-EOAD direction (Supplementary Table 1).

**FIGURE 1:**
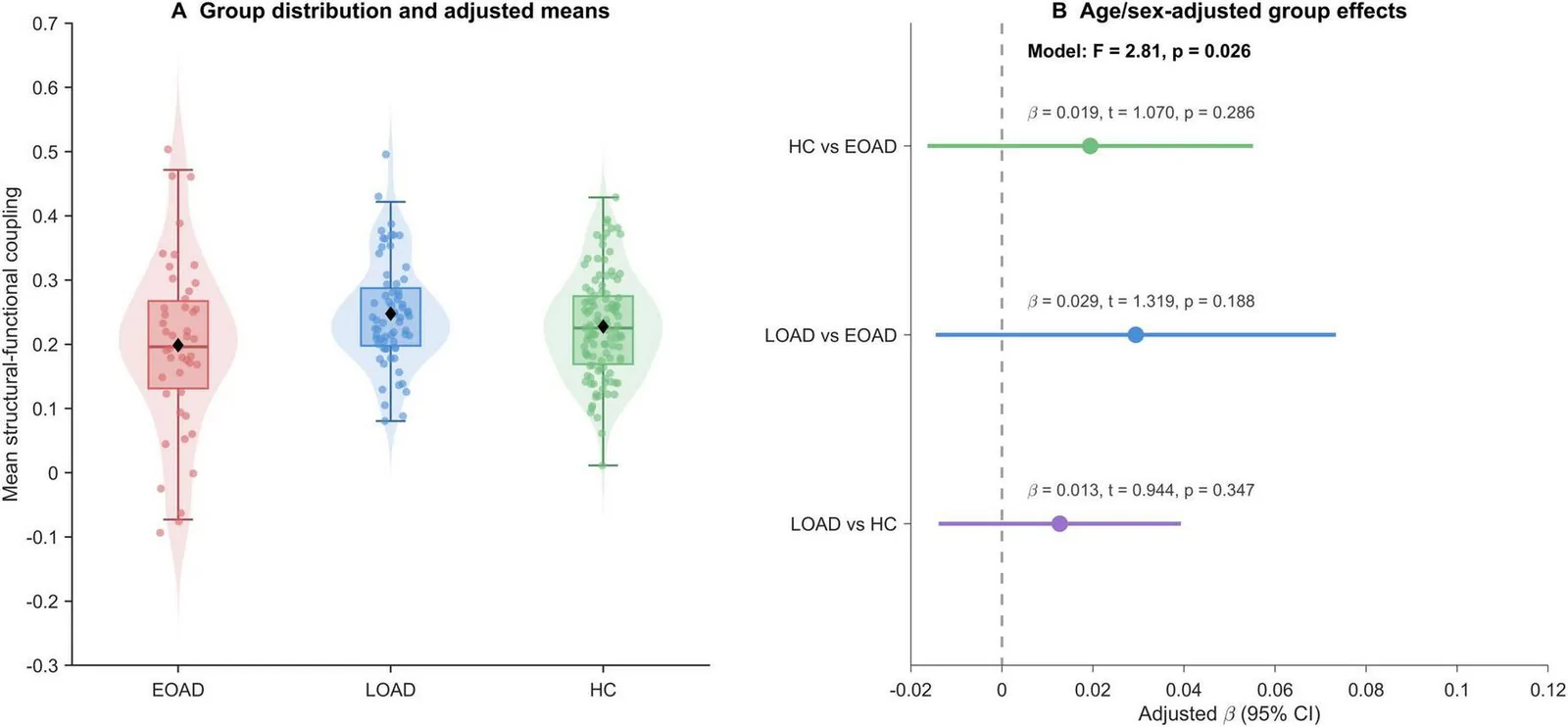
Global structural-functional coupling. **(A)** Violin/box plots showing group distributions of mean structural-functional coupling in the matched multimodal cohort. Diamonds indicate group means, and boxes indicate the interquartile range. **(B)** Age- and sex-adjusted pairwise group effects for HC vs. EOAD, LOAD vs. EOAD, and LOAD vs. HC. Points indicate adjusted β estimates, and horizontal lines indicate 95% confidence intervals. Positive β-values indicate higher mean coupling in the first-named group. Across all three pairwise contrasts, adjusted differences in global mean SFC were small and none reached statistical significance.

**FIGURE 2:**
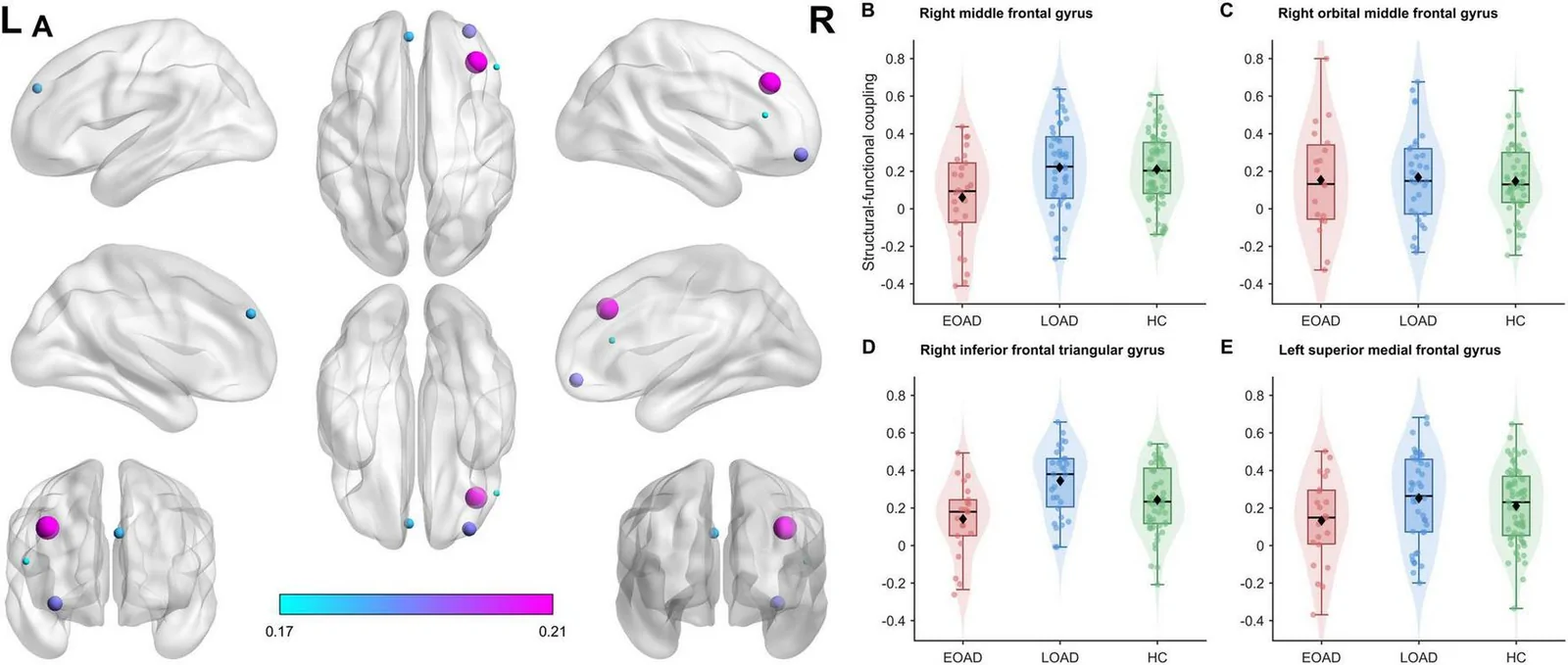
Regional frontal structural-functional coupling differences. **(A)** Surface rendering of the four frontal regions that survived FDR correction in the LOAD vs. EOAD comparison. **(B–E)** Group distributions for the right middle frontal gyrus, right orbital middle frontal gyrus, right inferior frontal triangular gyrus, and left superior medial frontal gyrus, arranged as a 2 × 2 block to the right of panel **(A)**. Violins show density, boxes indicate the interquartile range, and black diamonds indicate group means.

Together, these findings are consistent with an anatomically selective frontal coupling difference in the primary model rather than a generalized shift in mean structural-functional coupling (Figures 1, 2).

### 3.3 Graph-theoretical findings

**FIGURE 3:**
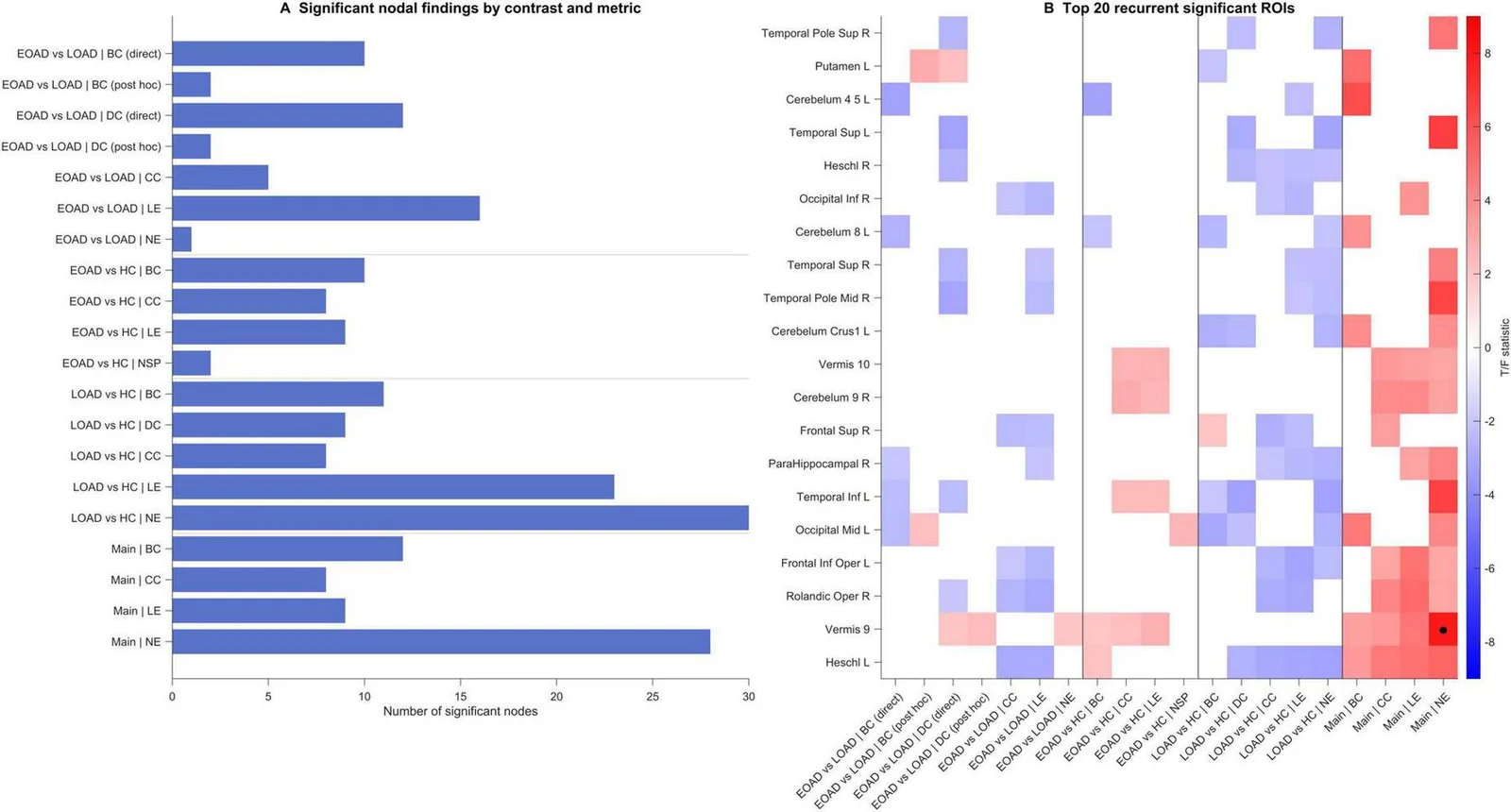
Graph-theoretical nodal summary. **(A)** Number of nominally significant nodal findings (raw *P* < 0.05) for each analysis-framework-by-contrast-by- metric combination. **(B)** Heatmap of the 20 most recurrent nominally significant nodal ROIs across combinations; colors indicate age- and sex-adjusted T statistics for pairwise contrasts and F statistics for the overall three-group effect. Blank cells indicate raw *P* ≥ 0.05, and dots indicate FDR *q* < 0.05 after Benjamini-Hochberg correction across 116 AAL nodes within the corresponding analysis framework, metric, and contrast. Only Vermis 9 nodal efficiency in the overall three-group analysis met this corrected threshold. Repeated contrast-by-metric labels correspond to distinct direct two-group and three-group *post hoc* workflows. Complete nominal node-level results are provided in Supplementary Table 3. BC, betweenness centrality; CC, nodal clustering coefficient; DC, degree centrality; EOAD, early-onset Alzheimer’s disease; HC, healthy controls; LE, nodal local efficiency; LOAD, late-onset Alzheimer’s disease; Main, overall three-group effect; NE, nodal efficiency; NSP, nodal shortest path length; ROI, region of interest.

Graph-theoretical analyses yielded numerous nominal node-level effects, but corrected evidence was limited. After age and sex adjustment and Benjamini-Hochberg correction across 116 nodes within each analysis-framework-by-metric-by-contrast family, only nodal efficiency in Vermis 9 showed a significant overall three-group effect (*F* = 8.031, *P* < 0.001, *q* = 0.049). No direct or post hoc EOAD-versus-LOAD nodal contrast survived FDR correction, and no disease-versus-control pairwise nodal contrast survived correction. Figure 3 displays the distribution of nominal effects and marks the single corrected result; Table 3 summarizes the analysis families with the largest numbers of nominally significant nodes. Complete nominal node-level results are provided in Supplementary Table 3.

**TABLE 3:** Summary of nodal graph-theoretical analysis families with at least 10 nominally significant nodes.

Metric	Contrast	No. of nominal nodes	Representative AAL region	*P*-value	FDR *q*-value
Nodal efficiency	LOAD vs. HC	30	Left Heschl gyrus	0.001	0.060
Nodal efficiency	Overall group effect	28	Vermis 9	<0.001	0.049*
Nodal local efficiency	LOAD vs. HC	23	Left inferior frontal opercular gyrus	0.002	0.095
Nodal local efficiency	EOAD vs. LOAD	16	Right rolandic operculum	0.003	0.177
Degree centrality	EOAD vs. LOAD	12	Left superior temporal gyrus	0.001	0.119
Betweenness centrality	Overall group effect	12	Left cerebellum lobules 4/5	0.002	0.263
Betweenness centrality	LOAD vs. HC	11	Left middle occipital gyrus	0.003	0.232
Betweenness centrality	EOAD vs. LOAD	10	Left cerebellum lobules 4/5	0.001	0.146
Betweenness centrality	EOAD vs. HC	10	Left cerebellum lobules 4/5	0.001	0.152

Counts refer to nodes with raw *P* < 0.05 and should not be interpreted as corrected discoveries. Rows are shown for analysis families with at least 10 nominally significant nodes. The representative region is the node with the smallest FDR *q*-value within each family. All graph models adjusted for age and sex. Benjamini-Hochberg FDR correction was applied separately across 116 AAL nodes within each analysis framework, metric, and contrast. Only the overall three-group effect in Vermis 9 nodal efficiency survived correction. Direct EOAD-versus-LOAD rows derive from the separate two-group workflow; overall and disease-versus-control rows derive from the three-group workflow. Complete nominal node-level results are reported in Supplementary Table 3. HC, healthy control; LOAD, late-onset Alzheimer’s disease; EOAD, early-onset Alzheimer’s disease.

*Indicates *q* < 0.05.

### 3.4 Clinical association analyses

Clinical association analyses were performed in the combined EOAD and LOAD sample for global mean SFC and the four principal frontal regional SFC measures across FAQ, MoCA, MMSE, GDS, CDR-SB, and ADAS, yielding 30 jointly corrected tests. No association reached nominal significance or survived Benjamini-Hochberg FDR correction. The smallest raw *P*-value was 0.118 for the association between FAQ and global mean SFC, with *q* = 0.929. Thus, the SFC measures did not show corrected associations with concurrent clinical variability (Supplementary Figure 2 and Supplementary Table 4).

### 3.5 Sensitivity analyses

Outlier removal produced nearly unchanged frontal coupling estimates. The positive LOAD-versus-EOAD pattern remained FDR-significant after adding education and after separately adding MMSE, MoCA, FAQ, GDS, CDR-SB, or ADAS total score. Atrophy-adjusted models preserved the positive direction across all four ROIs, with most effects remaining FDR-significant. Mean Power framewise displacement did not differ significantly among groups after age and sex adjustment, and all four frontal effects remained significant after its inclusion (all *q* = 0.031). Valid-edge count models were non-significant, and all four bootstrap confidence intervals excluded zero (Table 4, Supplementary Figure 3, and Supplementary Tables 1, 2, 5). These findings support conditional stability under these specifications but do not establish invariance across all modeling choices.

**TABLE 4:** FDR *q*-value summary of sensitivity analyses for the four frontal structural-functional coupling findings.

Model	Right middle frontal gyrus	Right orbital middle frontal gyrus	Right inferior frontal triangular gyrus	Left superior medial frontal gyrus
Age + sex	0.032*	0.032*	0.032*	0.032*
Age + sex + education	0.032*	0.032*	0.033*	0.033*
Age + sex + MMSE	0.032*	0.032*	0.032*	0.032*
Age + sex + MoCA	0.046*	0.046*	0.046*	0.046*
Age + sex + FAQ	0.037*	0.037*	0.037*	0.047*
Age + sex + GDS	0.030*	0.030*	0.038*	0.038*
Age + sex + CDR-SB	0.028*	0.028*	0.028*	0.028*
Age + sex + ADAS total score	0.027*	0.027*	0.033*	0.033*
Age + sex + hippocampal volume/eTIV	0.022*	0.014*	0.0497*	0.014*
Age + sex + hippocampal volume + eTIV	0.047*	0.012*	0.052	0.022*
Age + age^2^ + sex + education	0.063	0.042*	0.400	0.400
Age + sex + APOE ε4 carrier status	0.184	0.165	0.119	0.119
Age + sex + amyloid PET status	0.151	0.151	0.151	0.152
Age + sex + amyloid centiloids	0.149	0.130	0.130	0.149
AD-only: age + sex + disease stage	0.468	0.468	0.468	0.468
AD-only: overlap weighting	<0.001*	<0.001*	0.019*	0.001*
Age + sex + mean Power FD	0.031*	0.031*	0.031*	0.031*
Amyloid-negative HC restricted three-group model	0.047*	0.094	0.069	0.157

Cells report FDR *q*-values for the LOAD-versus-EOAD contrast; they are not effect-size estimates. *q*-values were corrected across 116 AAL regions within each model using the Benjamini-Hochberg procedure. The estimated direction was generally positive (higher SFC in LOAD), but statistical support varied by specification. Clinical covariates were entered separately. Models involving APOE ε4 carrier status, amyloid PET status, centiloids, disease stage, hippocampal atrophy, or mean Power FD used model- and ROI-specific complete cases, so *N* varied and is not represented by a single table-wide value. The overlap-weighted analysis used disease-only weights estimated from age, sex, and education. The amyloid-negative-control model retained all disease participants and restricted controls to participants with negative amyloid PET status. Complete model-specific estimates are reported in Supplementary Table 5; detailed ROI-specific coverage, motion results, and amyloid-negative-control contrasts are reported in Supplementary Tables 1, 2, AL, Automated Anatomical Labeling; ADAS, Alzheimer’s Disease Assessment Scale; APOE, apolipoprotein E; CDR-SB, Clinical Dementia Rating-Sum of Boxes; EOAD, early-onset Alzheimer’s disease; eTIV, estimated total intracranial volume; FAQ, Functional Activities Questionnaire; FDR, false discovery rate; GDS, Geriatric Depression Scale; LOAD, late-onset Alzheimer’s disease; MMSE, Mini-Mental State Examination; MoCA, Montreal Cognitive Assessment; PET, positron emission tomography; ROI, region of interest.

*Indicates *q* < 0.05.

Alternative age, disease-stage, biomarker, and sample-composition analyses provided more qualified support. In the quadratic-age model, all four estimates remained positive, but only the right orbital middle frontal region survived FDR correction (*q* = 0.042). Disease-only overlap weighting preserved all four corrected effects. In contrast, none of the four regions survived correction in the disease-only stage-adjusted model or in models additionally adjusted for APOE ε4 carrier status, amyloid PET status, or centiloids, although the estimated LOAD-versus-EOAD direction was generally positive. Restricting controls to 68 amyloid-negative participants retained positive estimates in all four regions, but only the right middle frontal region remained significant across 116 ROIs (*q* = 0.047), and no disease-versus-control regional contrast survived correction (Supplementary Tables 5, 6). These findings demonstrate model sensitivity and should not be attributed solely to smaller available-case samples.

### 3.6 Exploratory functional connectivity, local functional, structural decoupling, and DTI findings

Beyond the principal regional coupling and graph-theoretical results, global mean coupling, seed-based resting-state functional connectivity, local functional metrics, structural decoupling index, and DTI-derived regional measures were treated as exploratory or contextual components of the multimodal framework.

Seed-based resting-state functional connectivity showed voxel-level FDR-corrected clusters for EOAD versus healthy controls and LOAD versus healthy controls, but no EOAD-versus-LOAD cluster survived correction (Supplementary Table 7). Representative LOAD-versus-EOAD ALFF and fALFF estimates were heterogeneous and are presented descriptively as exploratory local-functional findings rather than primary subtype evidence (Supplementary Figures 4, 5 and Supplementary Table 8).

Structural decoupling index was successfully computed in the matched multimodal cohort, but no EOAD-versus-LOAD region survived FDR correction. Regional DTI-derived measures, including fractional anisotropy, mean diffusivity, axial diffusivity, radial diffusivity, and mode of anisotropy, likewise yielded no corrected EOAD-versus-LOAD finding. These domains were therefore less subtype-discriminative than frontal regional structural-functional coupling (Supplementary Figure 6).

## 4 Discussion

### 4.1 Principal findings

The principal finding was higher frontal regional structural-functional coupling in LOAD than EOAD in the right middle frontal, right orbital middle frontal, right inferior frontal triangular, and left superior medial frontal regions. This pattern occurred without a strong global mean coupling difference, indicating an anatomically selective rather than scalar subtype signal. In contrast, the graph-theoretical analysis did not provide broad corrected corroboration: only Vermis 9 nodal efficiency showed an overall group effect, and no EOAD-versus-LOAD nodal contrast survived FDR correction.

The frontal effects were directionally consistent across education, clinical-severity, hippocampal-atrophy, head-motion, and valid-edge analyses. All four remained FDR-significant after mean Power framewise displacement adjustment and under overlap weighting, and bootstrap confidence intervals excluded zero. These analyses make it less likely that the primary pattern is attributable only to broad clinical severity, gross hippocampal atrophy, head motion, or systematic differences in valid-edge coverage, while leaving other sources of confounding unresolved.

Nevertheless, corrected SFC evidence was substantially weaker under non-linear-age, disease-stage, biomarker-adjusted, and amyloid-negative-control specifications. In particular, only one region survived the quadratic-age model and none survived disease-stage, APOE ε4, amyloid PET, or centiloid adjustment. The result should therefore be framed as a model-sensitive frontal structure-function phenotype without corrected subtype-specific graph-theoretical corroboration, not as a definitive biomarker of symptom-onset biology or clinical subtype (Valdez-Gaxiola et al., 2024; Seath et al., 2024; Ottoy et al., 2025).

### 4.2 Interpretation of higher frontal structural-functional coupling in LOAD

Higher structural-functional coupling indicates closer statistical alignment between regional functional interactions and the estimated structural scaffold (Sun et al., 2024; Lu et al., 2025). The higher values observed in LOAD could reflect relative maintenance of structure-function correspondence, compensatory organization, reduced functional flexibility, aging-related network constraints, or methodological sensitivity to tractography and matrix construction. The cross-sectional design and model sensitivity prevent these possibilities from being distinguished, and higher coupling should not be interpreted as inherently beneficial or more preserved.

Conversely, lower frontal coupling in EOAD may be consistent with broader neocortical and association-network disruption reported in earlier-onset disease (Valdez-Gaxiola et al., 2024; Seath et al., 2024; Mehta et al., 2024; Katsumi et al., 2025). Higher coupling in LOAD should not automatically be interpreted as beneficial, because tighter dependence on direct structural pathways may also indicate reduced capacity for functional reconfiguration.

Persistence of the LOAD-versus-EOAD direction after hippocampal volume/eTIV adjustment suggests that the effect is not explained solely by head size or gross hippocampal atrophy. However, this adjustment does not capture cortical thinning, regional frontal atrophy, vascular injury, or other structural disease burden. The finding is therefore partly robust to atrophy adjustment but does not establish an atrophy-independent mechanism.

### 4.3 Relationship to healthy-control comparisons

Subtype comparisons and disease-versus-control comparisons address different questions. The absence of a corrected Alzheimer-versus-control regional effect does not negate a subtype difference within the Alzheimer spectrum, particularly when EOAD and LOAD may alter structure-function alignment in different anatomical distributions.

Spatially heterogeneous disease effects can weaken a pooled disease-versus-control contrast while producing a more coherent EOAD-versus-LOAD difference in selected frontal regions. The present results therefore support regional subtype heterogeneity rather than a monotonic disease-severity gradient.

Healthy-control comparisons remain contextual rather than diagnostic. Restricting controls to amyloid-negative participants did not produce corrected disease-versus-control regional effects and reduced LOAD-versus-EOAD support to the right middle frontal region alone, underscoring the influence of sample composition on whole-brain corrected inference.

### 4.4 Graph-theoretical and exploratory multimodal findings

Graph-theoretical findings were predominantly nominal. The only effect surviving within-family FDR correction was an overall three-group difference in Vermis 9 nodal efficiency; no EOAD-versus-LOAD nodal contrast survived correction. Because the surviving effect was omnibus rather than subtype-specific and arose in a single cerebellar vermis node, it should be interpreted cautiously and does not establish distributed topological separation between diagnosis-age-defined EOAD and LOAD.

The nominal graph patterns extended across several metrics and regions, but they remain descriptive because they did not survive node-wise correction. Graph measures are also sensitive to parcellation, tractography, thresholding, covariate specification, and network construction. Supplementary Table 3 separates direct two-group, three-group omnibus, and three-group post hoc workflows and reports raw *P* and FDR *q*-values, while regional structural-functional coupling remains the central positive imaging domain.

The exploratory analyses further clarify this hierarchy. Seed-based functional connectivity and local ALFF/fALFF measures documented functional abnormalities, whereas structural decoupling and regional DTI measures did not yield corrected EOAD-versus-LOAD effects. In this dataset, the clearest subtype signal therefore arose from regional structure-function coupling rather than nodal topology, average regional DTI properties, or graph-spectral decoupling alone (Gour et al., 2014; Adriaanse et al., 2014; Hrybouski et al., 2025; Preti and Van De Ville, 2019).

### 4.5 Robustness to age, sex, disease stage, and biomarker availability

Age and sex imbalance are inherent interpretive constraints because age defines the diagnostic grouping and sex distribution differed between groups. Primary covariate adjustment, non-linear-age modeling, overlap weighting, and interaction analyses were used to probe these influences, but their differing results show that statistical adjustment cannot recreate a naturally overlapping or balanced design or cleanly separate age-associated variation from diagnosis-age-defined subgroup effects.

Residual confounding by present age, vascular burden, scanner-related variability, or unmeasured aging factors therefore remains possible. The overlap-weighted and interaction analyses support the positive direction under selected specifications, whereas attenuation in the quadratic-age model requires continued caution.

Disease stage and biomarker specification also affected statistical support. Although estimates were generally positive, no frontal ROI survived whole-brain FDR correction after additional adjustment for MCI-versus-dementia stage, APOE ε4 carrier status, amyloid PET status, or centiloids. This loss of corrected support is evidence of model sensitivity and cannot be explained solely by reduced available-case sample size. Restricting controls to 68 amyloid-negative participants likewise retained support only in the right middle frontal region.

Available cognitive symptom-onset estimates supported the diagnosis-age-defined grouping: five EOAD and two LOAD participants lacked sufficient information, and no cross-group inconsistency was identified among the remainder. These retrospective ADNI estimates are not equivalent to prospectively adjudicated onset dates and should be interpreted accordingly (Petersen et al., 2010; Jack et al., 2008).

### 4.6 Clinical relevance

None of the 30 associations between the five SFC markers and six concurrent clinical scales reached nominal significance or survived joint FDR correction; the smallest raw *P*-value was 0.118 and the minimum *q*-value was 0.929. The frontal SFC phenotype therefore separated EOAD and LOAD more clearly than it explained cross-sectional variation in broad cognitive, functional, or affective scales.

Broad scales such as MMSE, MoCA, FAQ, GDS, CDR-SB, and ADAS may not capture the domain-specific cognitive or behavioral features most closely related to frontal network organization. Network markers may instead relate more strongly to longitudinal decline, cognitive profiles, or pathology distribution than to concurrent summary scores (Frontzkowski et al., 2022; Katsumi et al., 2025).

Accordingly, these findings do not establish diagnostic performance or clinical utility. Longitudinal and externally validated studies are needed to test whether frontal structural-functional coupling adds information beyond demographic, clinical, and biomarker variables or predicts subsequent decline (Ottoy et al., 2025).

### 4.7 Strengths and limitations

Strengths include integration of functional MRI, structural connectivity, regional structural-functional coupling, nodal graph metrics, structural decoupling, DTI-derived measures, clinical variables, biomarkers, and atrophy adjustment within one framework. The analysis distinguished principal from exploratory domains, applied feature-family correction, and reported both positive and negative results.

Several limitations remain. First, the study was cross-sectional and used a public multicenter cohort. Second, EOAD and LOAD were defined by age at first recorded diagnosis, with retrospective symptom-onset information available only for verification; this operational classification is not equivalent to prospectively adjudicated biological onset. Third, age separation and sex imbalance were inherent to the sample despite covariate, interaction, non-linear-age, and overlap-weighted analyses. Fourth, regional coupling used ROI-specific complete cases: total model samples were 195, 170, 177, and 176 across the four frontal ROIs, while calculable EOAD/LOAD counts were 41/61, 40/53, 38/59, and 36/55. Bootstrap results therefore support conditional stability among analyzable participants but do not remove uncertainty related to regional data availability.

Fifth, biomarker data were incomplete, and the clinically normal control group was not uniformly amyloid-negative; restriction to 68 amyloid-negative controls reduced corrected subtype support. Sixth, hippocampal volume/eTIV captures only part of structural disease burden. Seventh, the analysis used image-level ComBat harmonization with diagnostic group retained as a biological covariate, but a complete parallel reanalysis of all primary outcomes from unharmonized images was not available; dependence on the selected harmonization specification therefore cannot be excluded. Finally, coupling, graph, DTI, and local-functional findings may depend on AAL parcellation, linear atlas registration, probabilistic tractography settings, preprocessing, matrix normalization, and network-construction choices and require external replication.

### 4.8 Conclusion

In summary, the primary age- and sex-adjusted analysis showed higher frontal regional structural-functional coupling in diagnosis-age-defined LOAD than EOAD across four frontal regions. No EOAD-versus-LOAD nodal graph contrast survived within-family FDR correction. All four SFC effects survived motion adjustment and overlap weighting, but corrected support narrowed or disappeared under non-linear-age, disease-stage, biomarker-adjusted, and amyloid-negative-control specifications. Higher coupling cannot be assumed to indicate better-preserved function, and no SFC–clinical association survived joint FDR correction. The findings therefore support a model-sensitive frontal structure-function phenotype rather than a definitive onset-age biomarker or clinically validated subtype marker.

## Data Availability

The imaging and clinical data are available through the Alzheimer’s Disease Neuroimaging Initiative (ADNI) database (adni.loni.usc.edu), subject to ADNI data-access policies and approval. Custom Linux shell scripts and MATLAB code used for DTI processing, structural-connectivity reconstruction, coupling, structural decoupling, statistical analysis, and figure generation are available from the corresponding author upon reasonable request.

## Footnotes

1
https://adni.loni.usc.edu/
