# Supplementary material for: Multimodal network alterations in diagnosis-age-defined early- and late-onset Alzheimer’s disease
Source: Front Neurosci. 2026 Sep 8;20:1914358. doi: 10.3389/fnins.2026.1914358 (PMC13593455; doi:10.3389/fnins.2026.1914358)
Supplement: Supplementary file 1 [file Data_Sheet_1.docx]

**Supplementary Information**

1. **Supplementary figures**

**Supplementary Figure S1. Analytic cohort flow and ROI-specific sample coverage.**

*The functional MRI cohort included 50 participants with diagnosis-age-defined early-onset Alzheimer’s disease (EOAD), 72 with diagnosis-age-defined late-onset Alzheimer’s disease (LOAD), and 113 healthy controls (HC; total N=235). After exclusion of participants with unavailable or nonusable DTI or multimodal imaging data following modality-specific quality control (3 EOAD, 3 LOAD, and 2 HC), the matched multimodal cohort comprised 47 EOAD, 69 LOAD, and 111 HC participants (total N=227). Regional structural-functional coupling (SFC) sample sizes are shown for the right middle frontal gyrus, right orbital middle frontal gyrus, right inferior frontal triangular gyrus, and left superior medial frontal gyrus. Regional sample sizes differed because SFC was not calculable for every participant in every ROI. The ROI-specific samples were overlapping rather than mutually exclusive, and individual participants could contribute to more than one regional analysis. DTI, diffusion tensor imaging; ROI, region of interest.*

**Supplementary Figure S2. Clinical associations of structural-functional coupling markers with clinical scales.**

*Panels show age-, sex-, and subgroup-adjusted associations of mean SFC and four frontal regional SFC measures with six clinical scales in the combined EOAD/LOAD sample. Points and horizontal lines denote β estimates and 95% confidence intervals. No association reached nominal significance or survived joint Benjamini-Hochberg FDR correction across 30 tests.*

**Supplementary Figure S3. Outlier-removal sensitivity of structural-functional coupling effects.**

*A, adjusted LOAD-versus-EOAD estimates before and after excluding group-specific imaging values more than three standard deviations from the group mean for mean structural-functional coupling and the four frontal regional measures. Points indicate β estimates, and horizontal lines indicate 95% confidence intervals. B, corresponding significance profiles shown as -log10(P). Original models are shown in red and outlier-cleaned models in blue.*

**Supplementary Figure S4. Representative regional ALFF and fALFF effect estimates.**

*The upper portion shows representative ALFF effects and the lower portion representative fALFF effects for LOAD versus EOAD. Points indicate adjusted β estimates, and horizontal lines indicate 95% confidence intervals. Positive β values indicate higher signal in LOAD. These exploratory estimates are shown for context and are not interpreted as the primary subtype-discriminative evidence.*

**Supplementary Figure S5. Exploratory regional ALFF and fALFF displays.**

*A, ALFF regional display; B, fALFF regional display. Brain renderings show exploratory regions from the LOAD-versus-EOAD comparison, and color bars indicate signed adjusted group effects. These displays provide contextual local-functional findings and are not used as primary subtype-discriminative evidence.*

**Supplementary Figure S6. Representative structural-decoupling and DTI-derived regional effects.**

*A, structural decoupling index; B, fractional anisotropy; C, mean diffusivity. Points indicate adjusted β estimates for LOAD versus EOAD, and horizontal lines indicate 95% confidence intervals when available. These exploratory results document the structural-decoupling and DTI domains; no EOAD-versus-LOAD result in either domain survived FDR correction.*

**2. Supplementary tables**

**Supplementary Table S1. ROI-specific SFC calculability, valid-edge count models, and bootstrap stability for the four frontal findings.**

**Panel A. Regional SFC calculability and valid-edge distributions**

| **ROI** | **Group** | **Valid/total (%)** | **Median valid edges** | **IQR** | **Range** |
| --- | --- | --- | --- | --- | --- |
| Right middle frontal gyrus | EOAD | 41/47 (87.2%) | 78.0 | [60.2, 82.2] | 44-111 |
| Right middle frontal gyrus | LOAD | 61/69 (88.4%) | 60.0 | [54.0, 64.8] | 26-89 |
| Right middle frontal gyrus | HC | 93/111 (83.8%) | 58.0 | [51.0, 77.0] | 31-103 |
| Right orbital middle frontal gyrus | EOAD | 40/47 (85.1%) | 60.5 | [46.0, 66.0] | 11-103 |
| Right orbital middle frontal gyrus | LOAD | 53/69 (76.8%) | 54.0 | [49.2, 59.8] | 13-83 |
| Right orbital middle frontal gyrus | HC | 77/111 (69.4%) | 52.0 | [39.2, 57.2] | 8-69 |
| Right inferior frontal triangular gyrus | EOAD | 38/47 (80.9%) | 63.0 | [48.5, 80.0] | 11-110 |
| Right inferior frontal triangular gyrus | LOAD | 59/69 (85.5%) | 57.0 | [53.0, 62.2] | 13-88 |
| Right inferior frontal triangular gyrus | HC | 80/111 (72.1%) | 57.0 | [50.0, 75.0] | 7-99 |
| Left superior medial frontal gyrus | EOAD | 36/47 (76.6%) | 72.5 | [56.0, 88.0] | 24-113 |
| Left superior medial frontal gyrus | LOAD | 55/69 (79.7%) | 61.0 | [54.0, 69.0] | 17-88 |
| Left superior medial frontal gyrus | HC | 85/111 (76.6%) | 60.0 | [52.0, 80.0] | 16-103 |

**Panel B. LOAD-versus-EOAD valid-edge count models**

| **ROI** | **Model** | **N** | **β** | **95% CI** | **P value** | **FDR q value** |
| --- | --- | --- | --- | --- | --- | --- |
| Right middle frontal gyrus | Age + sex | 116 | -9.366 | [-30.585, 11.854] | 0.384 | 0.765 |
| Right middle frontal gyrus | Age + sex + ADNI phase | 116 | -11.023 | [-32.198, 10.153] | 0.304 | 0.609 |
| Right middle frontal gyrus | Age + sex + disease stage | 116 | -9.698 | [-30.725, 11.330] | 0.363 | 0.726 |
| Right orbital middle frontal gyrus | Age + sex | 116 | 3.265 | [-12.608, 19.138] | 0.684 | 0.765 |
| Right orbital middle frontal gyrus | Age + sex + ADNI phase | 116 | -0.417 | [-15.365, 14.532] | 0.956 | 0.956 |
| Right orbital middle frontal gyrus | Age + sex + disease stage | 116 | 3.161 | [-12.749, 19.072] | 0.695 | 0.744 |
| Right inferior frontal triangular gyrus | Age + sex | 116 | -3.090 | [-23.518, 17.338] | 0.765 | 0.765 |
| Right inferior frontal triangular gyrus | Age + sex + ADNI phase | 116 | -5.437 | [-25.828, 14.953] | 0.598 | 0.798 |
| Right inferior frontal triangular gyrus | Age + sex + disease stage | 116 | -3.356 | [-23.686, 16.974] | 0.744 | 0.744 |
| Left superior medial frontal gyrus | Age + sex | 116 | -12.952 | [-34.499, 8.596] | 0.236 | 0.765 |
| Left superior medial frontal gyrus | Age + sex + ADNI phase | 116 | -15.483 | [-36.762, 5.796] | 0.152 | 0.608 |
| Left superior medial frontal gyrus | Age + sex + disease stage | 116 | -13.301 | [-34.631, 8.030] | 0.219 | 0.726 |

**Panel C. Group-stratified bootstrap stability of LOAD-versus-EOAD frontal SFC effects**

| **ROI** | **EOAD/LOAD N** | **Original β** | **Bootstrap 95% CI** | **Positive direction** | **Bootstrap P value** | **FDR q value** |
| --- | --- | --- | --- | --- | --- | --- |
| Right middle frontal gyrus | 41/61 | 0.278 | [0.107, 0.464] | 99.84% | 0.003 | 0.021 |
| Right orbital middle frontal gyrus | 40/53 | 0.311 | [0.026, 0.564] | 98.18% | 0.036 | 0.024 |
| Right inferior frontal triangular gyrus | 38/59 | 0.264 | [0.106, 0.446] | 99.94% | 0.001 | 0.021 |
| Left superior medial frontal gyrus | 36/55 | 0.212 | [0.027, 0.403] | 98.64% | 0.027 | 0.024 |

***Footnote.*** *Regional SFC was defined as the Spearman correlation between log1p-transformed nonzero structural weights and the corresponding Fisher-z functional-connectivity values. The diagonal and nonfinite values were excluded, zero structural edges were omitted, negative functional-connectivity values were retained, and at least three valid edges were required. Panel A reports calculability and summarizes valid-edge counts among participants with calculable SFC. Panel B models valid-edge count, not coupling magnitude, in the combined EOAD and LOAD sample; N is the complete-case sample for each model, and q values were adjusted across the four frontal ROIs within each model. Panel C used 5,000 bootstrap repetitions stratified within EOAD and LOAD; EOAD/LOAD N gives the numbers of participants with calculable regional SFC in each group, so the results assess conditional stability among analyzable participants.*

**Supplementary Table S2. Mean Power framewise displacement and motion-adjusted structural-functional coupling sensitivity analyses.**

**Panel A. Descriptive group-level mean Power FD**

| **Group** | **N** | **Mean ± SD (mm)** | **Median (mm)** | **IQR** | **Range** |
| --- | --- | --- | --- | --- | --- |
| EOAD | 50 | 0.216 ± 0.143 | 0.163 | [0.117, 0.260] | [0.061, 0.645] |
| LOAD | 72 | 0.286 ± 0.208 | 0.233 | [0.144, 0.376] | [0.047, 1.313] |
| HC | 113 | 0.229 ± 0.150 | 0.213 | [0.106, 0.285] | [0.055, 0.786] |

**Panel B. Age- and sex-adjusted group comparisons of mean Power FD**

| **Contrast** | **N** | **β** | **95% CI** | **P value** | **BH q value** |
| --- | --- | --- | --- | --- | --- |
| LOAD vs EOAD | 122 | 0.067 | [-0.042, 0.177] | 0.227 | 0.340 |
| HC vs EOAD | 163 | -0.011 | [-0.069, 0.046] | 0.697 | 0.697 |
| HC vs LOAD | 185 | -0.052 | [-0.108, 0.004] | 0.068 | 0.203 |

**Panel C. LOAD-versus-EOAD frontal SFC effects after additional mean Power FD adjustment**

| **ROI** | **Total complete-case N** | **β** | **95% CI** | **P value** | **FDR q value** |
| --- | --- | --- | --- | --- | --- |
| Right middle frontal gyrus | 195 | 0.258 | [0.115, 0.400] | <0.001 | 0.031 |
| Right orbital middle frontal gyrus | 170 | 0.316 | [0.136, 0.496] | <0.001 | 0.031 |
| Right inferior frontal triangular gyrus | 177 | 0.242 | [0.101, 0.383] | <0.001 | 0.031 |
| Left superior medial frontal gyrus | 176 | 0.220 | [0.090, 0.349] | 0.001 | 0.031 |

***Footnote.*** *Mean Power FD was derived from DPABI realignment parameters. Panel A provides descriptive group summaries; the age- and sex-adjusted omnibus group effect was P = 0.194, and no q value was calculated because this was a single test. Panel B reports pairwise adjusted P values and Benjamini-Hochberg q values across the three contrasts. Panel C models included EOAD, LOAD, and healthy controls and adjusted for age, sex, and mean Power FD; N is the total ROI-specific complete-case sample across all three groups. Panel C q values were corrected across 116 AAL regions for the LOAD-versus-EOAD contrast.*

**Supplementary Table S3. Complete nominal nodal graph-theoretical findings with within-family FDR correction.**

| **Analysis framework** | **Metric** | **Contrast** | **AAL region label** | **T/F statistic** | **P value** | **FDR q value** |
| --- | --- | --- | --- | --- | --- | --- |
| Direct two-group | Betweenness centrality | EOAD vs LOAD | Frontal Mid R | 2.886 | 0.004 | 0.248 |
| Direct two-group | Betweenness centrality | EOAD vs LOAD | Supp Motor Area R | 2.103 | 0.037 | 0.501 |
| Direct two-group | Betweenness centrality | EOAD vs LOAD | ParaHippocampal R | -1.978 | 0.049 | 0.570 |
| Direct two-group | Betweenness centrality | EOAD vs LOAD | Occipital Mid L | -2.418 | 0.016 | 0.357 |
| Direct two-group | Betweenness centrality | EOAD vs LOAD | Precuneus R | 2.077 | 0.039 | 0.501 |
| Direct two-group | Betweenness centrality | EOAD vs LOAD | Pallidum L | -2.373 | 0.018 | 0.357 |
| Direct two-group | Betweenness centrality | EOAD vs LOAD | Temporal Inf L | -2.375 | 0.018 | 0.357 |
| Direct two-group | Betweenness centrality | EOAD vs LOAD | Cerebelum 4 5 L | -3.266 | 0.001 | 0.146 |
| Direct two-group | Betweenness centrality | EOAD vs LOAD | Cerebelum 6 L | -2.099 | 0.037 | 0.501 |
| Direct two-group | Betweenness centrality | EOAD vs LOAD | Cerebelum 8 L | -2.740 | 0.007 | 0.256 |
| Direct two-group | Degree centrality | EOAD vs LOAD | Rolandic Oper R | -1.995 | 0.047 | 0.403 |
| Direct two-group | Degree centrality | EOAD vs LOAD | Cuneus L | -2.583 | 0.010 | 0.226 |
| Direct two-group | Degree centrality | EOAD vs LOAD | Cuneus R | -2.145 | 0.033 | 0.383 |
| Direct two-group | Degree centrality | EOAD vs LOAD | Putamen L | 2.183 | 0.030 | 0.383 |
| Direct two-group | Degree centrality | EOAD vs LOAD | Heschl R | -2.709 | 0.007 | 0.226 |
| Direct two-group | Degree centrality | EOAD vs LOAD | Temporal Sup L | -3.303 | 0.001 | 0.119 |
| Direct two-group | Degree centrality | EOAD vs LOAD | Temporal Sup R | -2.648 | 0.009 | 0.226 |
| Direct two-group | Degree centrality | EOAD vs LOAD | Temporal Pole Sup R | -2.541 | 0.012 | 0.226 |
| Direct two-group | Degree centrality | EOAD vs LOAD | Temporal Pole Mid L | -2.373 | 0.018 | 0.268 |
| Direct two-group | Degree centrality | EOAD vs LOAD | Temporal Pole Mid R | -3.117 | 0.002 | 0.119 |
| Direct two-group | Degree centrality | EOAD vs LOAD | Temporal Inf L | -2.372 | 0.019 | 0.268 |
| Direct two-group | Degree centrality | EOAD vs LOAD | Vermis 9 | 2.041 | 0.042 | 0.403 |
| Direct two-group | Nodal clustering coefficient | EOAD vs LOAD | Frontal Sup R | -2.413 | 0.017 | 0.643 |
| Direct two-group | Nodal clustering coefficient | EOAD vs LOAD | Frontal Inf Oper L | -1.992 | 0.048 | 0.764 |
| Direct two-group | Nodal clustering coefficient | EOAD vs LOAD | Rolandic Oper R | -2.653 | 0.009 | 0.495 |
| Direct two-group | Nodal clustering coefficient | EOAD vs LOAD | Occipital Inf R | -2.070 | 0.040 | 0.764 |
| Direct two-group | Nodal clustering coefficient | EOAD vs LOAD | Heschl L | -2.959 | 0.003 | 0.396 |
| Direct two-group | Nodal local efficiency | EOAD vs LOAD | Frontal Sup R | -2.385 | 0.018 | 0.297 |
| Direct two-group | Nodal local efficiency | EOAD vs LOAD | Frontal Inf Oper L | -2.572 | 0.011 | 0.297 |
| Direct two-group | Nodal local efficiency | EOAD vs LOAD | Frontal Inf Tri R | -1.985 | 0.048 | 0.325 |
| Direct two-group | Nodal local efficiency | EOAD vs LOAD | Frontal Inf Orb L | -2.256 | 0.025 | 0.299 |
| Direct two-group | Nodal local efficiency | EOAD vs LOAD | Frontal Inf Orb R | -2.390 | 0.018 | 0.297 |
| Direct two-group | Nodal local efficiency | EOAD vs LOAD | Rolandic Oper R | -2.994 | 0.003 | 0.177 |
| Direct two-group | Nodal local efficiency | EOAD vs LOAD | Supp Motor Area R | -2.059 | 0.041 | 0.323 |
| Direct two-group | Nodal local efficiency | EOAD vs LOAD | Frontal Sup Medial L | -2.206 | 0.028 | 0.299 |
| Direct two-group | Nodal local efficiency | EOAD vs LOAD | Frontal Sup Medial R | -2.047 | 0.042 | 0.323 |
| Direct two-group | Nodal local efficiency | EOAD vs LOAD | Cingulum Mid R | -2.229 | 0.027 | 0.299 |
| Direct two-group | Nodal local efficiency | EOAD vs LOAD | ParaHippocampal R | -2.090 | 0.038 | 0.323 |
| Direct two-group | Nodal local efficiency | EOAD vs LOAD | Occipital Sup R | -2.248 | 0.026 | 0.299 |
| Direct two-group | Nodal local efficiency | EOAD vs LOAD | Occipital Inf R | -2.559 | 0.011 | 0.297 |
| Direct two-group | Nodal local efficiency | EOAD vs LOAD | Heschl L | -3.013 | 0.003 | 0.177 |
| Direct two-group | Nodal local efficiency | EOAD vs LOAD | Temporal Sup R | -2.151 | 0.032 | 0.314 |
| Direct two-group | Nodal local efficiency | EOAD vs LOAD | Temporal Pole Mid R | -2.444 | 0.015 | 0.297 |
| Three-group omnibus | Betweenness centrality | Overall group effect | Frontal Mid R | 4.272 | 0.015 | 0.341 |
| Three-group omnibus | Betweenness centrality | Overall group effect | Amygdala R | 3.175 | 0.044 | 0.422 |
| Three-group omnibus | Betweenness centrality | Overall group effect | Occipital Mid L | 4.697 | 0.010 | 0.341 |
| Three-group omnibus | Betweenness centrality | Overall group effect | Precuneus R | 3.982 | 0.020 | 0.341 |
| Three-group omnibus | Betweenness centrality | Overall group effect | Caudate L | 3.691 | 0.026 | 0.341 |
| Three-group omnibus | Betweenness centrality | Overall group effect | Caudate R | 3.828 | 0.023 | 0.341 |
| Three-group omnibus | Betweenness centrality | Overall group effect | Putamen L | 5.113 | 0.007 | 0.341 |
| Three-group omnibus | Betweenness centrality | Overall group effect | Heschl L | 3.561 | 0.030 | 0.348 |
| Three-group omnibus | Betweenness centrality | Overall group effect | Cerebelum Crus1 L | 4.015 | 0.019 | 0.341 |
| Three-group omnibus | Betweenness centrality | Overall group effect | Cerebelum 4 5 L | 6.253 | 0.002 | 0.263 |
| Three-group omnibus | Betweenness centrality | Overall group effect | Cerebelum 8 L | 3.797 | 0.024 | 0.341 |
| Three-group omnibus | Betweenness centrality | Overall group effect | Vermis 9 | 3.367 | 0.036 | 0.382 |
| Three-group post hoc | Betweenness centrality | EOAD vs LOAD | Occipital Mid L | 2.182 | 0.031 | 0.947 |
| Three-group post hoc | Betweenness centrality | EOAD vs LOAD | Putamen L | 2.939 | 0.004 | 0.459 |
| Three-group post hoc | Betweenness centrality | EOAD vs HC | Frontal Mid R | 2.276 | 0.024 | 0.490 |
| Three-group post hoc | Betweenness centrality | EOAD vs HC | Insula R | 2.397 | 0.018 | 0.490 |
| Three-group post hoc | Betweenness centrality | EOAD vs HC | Amygdala R | -2.499 | 0.013 | 0.490 |
| Three-group post hoc | Betweenness centrality | EOAD vs HC | Occipital Mid R | 2.258 | 0.025 | 0.490 |
| Three-group post hoc | Betweenness centrality | EOAD vs HC | Paracentral Lobule L | 2.660 | 0.009 | 0.490 |
| Three-group post hoc | Betweenness centrality | EOAD vs HC | Pallidum L | -2.079 | 0.039 | 0.521 |
| Three-group post hoc | Betweenness centrality | EOAD vs HC | Heschl L | 2.145 | 0.033 | 0.521 |
| Three-group post hoc | Betweenness centrality | EOAD vs HC | Cerebelum 4 5 L | -3.271 | 0.001 | 0.152 |
| Three-group post hoc | Betweenness centrality | EOAD vs HC | Cerebelum 8 L | -2.041 | 0.043 | 0.521 |
| Three-group post hoc | Betweenness centrality | EOAD vs HC | Vermis 9 | 2.021 | 0.045 | 0.521 |
| Three-group post hoc | Betweenness centrality | LOAD vs HC | Frontal Sup R | 2.107 | 0.036 | 0.456 |
| Three-group post hoc | Betweenness centrality | LOAD vs HC | Frontal Mid R | 2.293 | 0.023 | 0.456 |
| Three-group post hoc | Betweenness centrality | LOAD vs HC | Insula L | -2.183 | 0.030 | 0.456 |
| Three-group post hoc | Betweenness centrality | LOAD vs HC | Occipital Mid L | -3.051 | 0.003 | 0.232 |
| Three-group post hoc | Betweenness centrality | LOAD vs HC | Precuneus R | 2.781 | 0.006 | 0.232 |
| Three-group post hoc | Betweenness centrality | LOAD vs HC | Caudate L | -2.243 | 0.026 | 0.456 |
| Three-group post hoc | Betweenness centrality | LOAD vs HC | Putamen L | -2.076 | 0.039 | 0.456 |
| Three-group post hoc | Betweenness centrality | LOAD vs HC | Temporal Inf L | -2.004 | 0.047 | 0.491 |
| Three-group post hoc | Betweenness centrality | LOAD vs HC | Cerebelum Crus1 L | -2.784 | 0.006 | 0.232 |
| Three-group post hoc | Betweenness centrality | LOAD vs HC | Cerebelum 6 L | -2.099 | 0.037 | 0.456 |
| Three-group post hoc | Betweenness centrality | LOAD vs HC | Cerebelum 8 L | -2.526 | 0.012 | 0.360 |
| Three-group post hoc | Degree centrality | EOAD vs LOAD | Angular L | 1.989 | 0.049 | 0.801 |
| Three-group post hoc | Degree centrality | EOAD vs LOAD | Vermis 9 | 2.386 | 0.019 | 0.801 |
| Three-group post hoc | Degree centrality | LOAD vs HC | Insula L | -2.399 | 0.017 | 0.289 |
| Three-group post hoc | Degree centrality | LOAD vs HC | Occipital Mid L | -2.245 | 0.026 | 0.335 |
| Three-group post hoc | Degree centrality | LOAD vs HC | Thalamus R | 2.491 | 0.014 | 0.263 |
| Three-group post hoc | Degree centrality | LOAD vs HC | Heschl L | -2.713 | 0.007 | 0.245 |
| Three-group post hoc | Degree centrality | LOAD vs HC | Heschl R | -2.586 | 0.010 | 0.245 |
| Three-group post hoc | Degree centrality | LOAD vs HC | Temporal Sup L | -2.913 | 0.004 | 0.234 |
| Three-group post hoc | Degree centrality | LOAD vs HC | Temporal Pole Sup R | -2.260 | 0.025 | 0.335 |
| Three-group post hoc | Degree centrality | LOAD vs HC | Temporal Inf L | -3.261 | 0.001 | 0.154 |
| Three-group post hoc | Degree centrality | LOAD vs HC | Cerebelum Crus1 L | -2.584 | 0.011 | 0.245 |
| Three-group omnibus | Nodal clustering coefficient | Overall group effect | Frontal Sup R | 3.394 | 0.035 | 0.652 |
| Three-group omnibus | Nodal clustering coefficient | Overall group effect | Frontal Inf Oper L | 3.099 | 0.047 | 0.652 |
| Three-group omnibus | Nodal clustering coefficient | Overall group effect | Rolandic Oper R | 4.237 | 0.016 | 0.652 |
| Three-group omnibus | Nodal clustering coefficient | Overall group effect | Heschl L | 4.687 | 0.010 | 0.652 |
| Three-group omnibus | Nodal clustering coefficient | Overall group effect | Cerebelum 9 R | 4.064 | 0.018 | 0.652 |
| Three-group omnibus | Nodal clustering coefficient | Overall group effect | Cerebelum 10 R | 3.232 | 0.041 | 0.652 |
| Three-group omnibus | Nodal clustering coefficient | Overall group effect | Vermis 9 | 3.552 | 0.030 | 0.652 |
| Three-group omnibus | Nodal clustering coefficient | Overall group effect | Vermis 10 | 3.645 | 0.028 | 0.652 |
| Three-group post hoc | Nodal clustering coefficient | EOAD vs HC | Occipital Mid R | -2.177 | 0.031 | 0.513 |
| Three-group post hoc | Nodal clustering coefficient | EOAD vs HC | Paracentral Lobule L | -2.467 | 0.015 | 0.363 |
| Three-group post hoc | Nodal clustering coefficient | EOAD vs HC | Temporal Inf L | 2.444 | 0.016 | 0.363 |
| Three-group post hoc | Nodal clustering coefficient | EOAD vs HC | Cerebelum 9 R | 2.912 | 0.004 | 0.363 |
| Three-group post hoc | Nodal clustering coefficient | EOAD vs HC | Cerebelum 10 L | 2.591 | 0.010 | 0.363 |
| Three-group post hoc | Nodal clustering coefficient | EOAD vs HC | Vermis 8 | 2.056 | 0.041 | 0.600 |
| Three-group post hoc | Nodal clustering coefficient | EOAD vs HC | Vermis 9 | 2.305 | 0.022 | 0.434 |
| Three-group post hoc | Nodal clustering coefficient | EOAD vs HC | Vermis 10 | 2.659 | 0.009 | 0.363 |
| Three-group post hoc | Nodal clustering coefficient | LOAD vs HC | Frontal Sup R | -2.794 | 0.006 | 0.223 |
| Three-group post hoc | Nodal clustering coefficient | LOAD vs HC | Frontal Inf Oper L | -2.666 | 0.008 | 0.243 |
| Three-group post hoc | Nodal clustering coefficient | LOAD vs HC | Rolandic Oper R | -2.932 | 0.004 | 0.220 |
| Three-group post hoc | Nodal clustering coefficient | LOAD vs HC | Frontal Sup Medial R | -2.056 | 0.041 | 0.535 |
| Three-group post hoc | Nodal clustering coefficient | LOAD vs HC | ParaHippocampal R | -2.079 | 0.039 | 0.535 |
| Three-group post hoc | Nodal clustering coefficient | LOAD vs HC | Occipital Inf R | -2.111 | 0.036 | 0.535 |
| Three-group post hoc | Nodal clustering coefficient | LOAD vs HC | Heschl L | -3.044 | 0.003 | 0.220 |
| Three-group post hoc | Nodal clustering coefficient | LOAD vs HC | Heschl R | -2.127 | 0.035 | 0.535 |
| Three-group omnibus | Nodal efficiency | Overall group effect | Frontal Inf Oper L | 3.065 | 0.049 | 0.195 |
| Three-group omnibus | Nodal efficiency | Overall group effect | Frontal Inf Tri R | 3.138 | 0.045 | 0.195 |
| Three-group omnibus | Nodal efficiency | Overall group effect | Frontal Inf Orb R | 3.042 | 0.050 | 0.195 |
| Three-group omnibus | Nodal efficiency | Overall group effect | Rolandic Oper R | 3.154 | 0.045 | 0.195 |
| Three-group omnibus | Nodal efficiency | Overall group effect | Insula L | 3.603 | 0.029 | 0.195 |
| Three-group omnibus | Nodal efficiency | Overall group effect | ParaHippocampal L | 3.045 | 0.050 | 0.195 |
| Three-group omnibus | Nodal efficiency | Overall group effect | ParaHippocampal R | 4.248 | 0.015 | 0.176 |
| Three-group omnibus | Nodal efficiency | Overall group effect | Cuneus L | 4.555 | 0.011 | 0.157 |
| Three-group omnibus | Nodal efficiency | Overall group effect | Cuneus R | 3.710 | 0.026 | 0.195 |
| Three-group omnibus | Nodal efficiency | Overall group effect | Occipital Sup L | 3.799 | 0.024 | 0.195 |
| Three-group omnibus | Nodal efficiency | Overall group effect | Occipital Mid L | 4.169 | 0.017 | 0.176 |
| Three-group omnibus | Nodal efficiency | Overall group effect | Occipital Inf L | 3.746 | 0.025 | 0.195 |
| Three-group omnibus | Nodal efficiency | Overall group effect | Fusiform L | 3.550 | 0.030 | 0.195 |
| Three-group omnibus | Nodal efficiency | Overall group effect | Postcentral L | 3.046 | 0.049 | 0.195 |
| Three-group omnibus | Nodal efficiency | Overall group effect | Angular L | 3.207 | 0.042 | 0.195 |
| Three-group omnibus | Nodal efficiency | Overall group effect | Heschl L | 5.380 | 0.005 | 0.118 |
| Three-group omnibus | Nodal efficiency | Overall group effect | Temporal Sup L | 6.714 | 0.001 | 0.052 |
| Three-group omnibus | Nodal efficiency | Overall group effect | Temporal Sup R | 4.490 | 0.012 | 0.157 |
| Three-group omnibus | Nodal efficiency | Overall group effect | Temporal Pole Sup L | 3.419 | 0.034 | 0.195 |
| Three-group omnibus | Nodal efficiency | Overall group effect | Temporal Pole Sup R | 4.730 | 0.010 | 0.157 |
| Three-group omnibus | Nodal efficiency | Overall group effect | Temporal Mid L | 3.400 | 0.035 | 0.195 |
| Three-group omnibus | Nodal efficiency | Overall group effect | Temporal Pole Mid L | 5.213 | 0.006 | 0.118 |
| Three-group omnibus | Nodal efficiency | Overall group effect | Temporal Pole Mid R | 6.507 | 0.002 | 0.052 |
| Three-group omnibus | Nodal efficiency | Overall group effect | Temporal Inf L | 6.639 | 0.002 | 0.052 |
| Three-group omnibus | Nodal efficiency | Overall group effect | Cerebelum Crus1 L | 3.886 | 0.022 | 0.195 |
| Three-group omnibus | Nodal efficiency | Overall group effect | Cerebelum 9 R | 3.240 | 0.041 | 0.195 |
| Three-group omnibus | Nodal efficiency | Overall group effect | Vermis 9 | **8.031** | **<0.001** | **0.049** |
| Three-group omnibus | Nodal efficiency | Overall group effect | Vermis 10 | 3.126 | 0.046 | 0.195 |
| Three-group post hoc | Nodal efficiency | EOAD vs LOAD | Vermis 9 | 2.047 | 0.043 | 0.996 |
| Three-group post hoc | Nodal efficiency | LOAD vs HC | Frontal Inf Oper L | -2.345 | 0.020 | 0.146 |
| Three-group post hoc | Nodal efficiency | LOAD vs HC | Frontal Inf Tri L | -2.115 | 0.036 | 0.158 |
| Three-group post hoc | Nodal efficiency | LOAD vs HC | Frontal Inf Tri R | -2.507 | 0.013 | 0.138 |
| Three-group post hoc | Nodal efficiency | LOAD vs HC | Frontal Inf Orb R | -2.238 | 0.026 | 0.146 |
| Three-group post hoc | Nodal efficiency | LOAD vs HC | Insula L | -2.612 | 0.010 | 0.138 |
| Three-group post hoc | Nodal efficiency | LOAD vs HC | ParaHippocampal L | -2.230 | 0.027 | 0.146 |
| Three-group post hoc | Nodal efficiency | LOAD vs HC | ParaHippocampal R | -2.692 | 0.008 | 0.138 |
| Three-group post hoc | Nodal efficiency | LOAD vs HC | Amygdala R | -2.142 | 0.034 | 0.156 |
| Three-group post hoc | Nodal efficiency | LOAD vs HC | Cuneus L | -2.376 | 0.019 | 0.146 |
| Three-group post hoc | Nodal efficiency | LOAD vs HC | Cuneus R | -2.219 | 0.028 | 0.146 |
| Three-group post hoc | Nodal efficiency | LOAD vs HC | Occipital Sup L | -2.475 | 0.014 | 0.138 |
| Three-group post hoc | Nodal efficiency | LOAD vs HC | Occipital Mid L | -2.732 | 0.007 | 0.138 |
| Three-group post hoc | Nodal efficiency | LOAD vs HC | Occipital Inf L | -2.575 | 0.011 | 0.138 |
| Three-group post hoc | Nodal efficiency | LOAD vs HC | Fusiform L | -2.484 | 0.014 | 0.138 |
| Three-group post hoc | Nodal efficiency | LOAD vs HC | Postcentral L | -2.059 | 0.041 | 0.158 |
| Three-group post hoc | Nodal efficiency | LOAD vs HC | SupraMarginal L | -2.162 | 0.032 | 0.154 |
| Three-group post hoc | Nodal efficiency | LOAD vs HC | Heschl L | -3.290 | 0.001 | 0.060 |
| Three-group post hoc | Nodal efficiency | LOAD vs HC | Heschl R | -2.289 | 0.023 | 0.146 |
| Three-group post hoc | Nodal efficiency | LOAD vs HC | Temporal Sup L | -3.211 | 0.002 | 0.060 |
| Three-group post hoc | Nodal efficiency | LOAD vs HC | Temporal Sup R | -2.268 | 0.025 | 0.146 |
| Three-group post hoc | Nodal efficiency | LOAD vs HC | Temporal Pole Sup L | -2.234 | 0.027 | 0.146 |
| Three-group post hoc | Nodal efficiency | LOAD vs HC | Temporal Pole Sup R | -2.697 | 0.008 | 0.138 |
| Three-group post hoc | Nodal efficiency | LOAD vs HC | Temporal Mid L | -2.343 | 0.020 | 0.146 |
| Three-group post hoc | Nodal efficiency | LOAD vs HC | Temporal Pole Mid L | -2.178 | 0.031 | 0.154 |
| Three-group post hoc | Nodal efficiency | LOAD vs HC | Temporal Pole Mid R | -2.378 | 0.018 | 0.146 |
| Three-group post hoc | Nodal efficiency | LOAD vs HC | Temporal Inf L | -3.242 | 0.001 | 0.060 |
| Three-group post hoc | Nodal efficiency | LOAD vs HC | Cerebelum Crus1 L | -2.654 | 0.009 | 0.138 |
| Three-group post hoc | Nodal efficiency | LOAD vs HC | Cerebelum Crus1 R | -2.066 | 0.040 | 0.158 |
| Three-group post hoc | Nodal efficiency | LOAD vs HC | Cerebelum 6 L | -2.071 | 0.040 | 0.158 |
| Three-group post hoc | Nodal efficiency | LOAD vs HC | Cerebelum 8 L | -2.059 | 0.041 | 0.158 |
| Three-group omnibus | Nodal local efficiency | Overall group effect | Frontal Inf Oper L | 4.884 | 0.008 | 0.284 |
| Three-group omnibus | Nodal local efficiency | Overall group effect | Rolandic Oper R | 5.202 | 0.006 | 0.284 |
| Three-group omnibus | Nodal local efficiency | Overall group effect | Frontal Sup Medial L | 3.142 | 0.045 | 0.367 |
| Three-group omnibus | Nodal local efficiency | Overall group effect | ParaHippocampal R | 3.213 | 0.042 | 0.367 |
| Three-group omnibus | Nodal local efficiency | Overall group effect | Occipital Inf R | 3.783 | 0.024 | 0.367 |
| Three-group omnibus | Nodal local efficiency | Overall group effect | Heschl L | 4.985 | 0.008 | 0.284 |
| Three-group omnibus | Nodal local efficiency | Overall group effect | Cerebelum 9 R | 4.113 | 0.018 | 0.367 |
| Three-group omnibus | Nodal local efficiency | Overall group effect | Vermis 9 | 4.721 | 0.010 | 0.284 |
| Three-group omnibus | Nodal local efficiency | Overall group effect | Vermis 10 | 3.407 | 0.035 | 0.367 |
| Three-group post hoc | Nodal local efficiency | EOAD vs HC | Frontal Inf Orb R | -2.044 | 0.043 | 0.550 |
| Three-group post hoc | Nodal local efficiency | EOAD vs HC | Occipital Mid R | -2.163 | 0.032 | 0.466 |
| Three-group post hoc | Nodal local efficiency | EOAD vs HC | Paracentral Lobule L | -2.531 | 0.012 | 0.286 |
| Three-group post hoc | Nodal local efficiency | EOAD vs HC | Temporal Inf L | 2.439 | 0.016 | 0.306 |
| Three-group post hoc | Nodal local efficiency | EOAD vs HC | Cerebelum 9 R | 2.549 | 0.012 | 0.286 |
| Three-group post hoc | Nodal local efficiency | EOAD vs HC | Cerebelum 10 L | 2.644 | 0.009 | 0.286 |
| Three-group post hoc | Nodal local efficiency | EOAD vs HC | Vermis 8 | 2.162 | 0.032 | 0.466 |
| Three-group post hoc | Nodal local efficiency | EOAD vs HC | Vermis 9 | 2.796 | 0.006 | 0.286 |
| Three-group post hoc | Nodal local efficiency | EOAD vs HC | Vermis 10 | 2.672 | 0.008 | 0.286 |
| Three-group post hoc | Nodal local efficiency | LOAD vs HC | Frontal Sup R | -2.325 | 0.021 | 0.245 |
| Three-group post hoc | Nodal local efficiency | LOAD vs HC | Frontal Inf Oper L | -3.196 | 0.002 | 0.095 |
| Three-group post hoc | Nodal local efficiency | LOAD vs HC | Frontal Inf Oper R | -2.001 | 0.047 | 0.245 |
| Three-group post hoc | Nodal local efficiency | LOAD vs HC | Frontal Inf Tri L | -2.077 | 0.039 | 0.245 |
| Three-group post hoc | Nodal local efficiency | LOAD vs HC | Frontal Inf Tri R | -2.390 | 0.018 | 0.245 |
| Three-group post hoc | Nodal local efficiency | LOAD vs HC | Frontal Inf Orb L | -2.205 | 0.029 | 0.245 |
| Three-group post hoc | Nodal local efficiency | LOAD vs HC | Rolandic Oper R | -3.073 | 0.002 | 0.095 |
| Three-group post hoc | Nodal local efficiency | LOAD vs HC | Supp Motor Area R | -1.986 | 0.049 | 0.245 |
| Three-group post hoc | Nodal local efficiency | LOAD vs HC | Frontal Sup Medial L | -2.270 | 0.024 | 0.245 |
| Three-group post hoc | Nodal local efficiency | LOAD vs HC | Frontal Sup Medial R | -2.127 | 0.035 | 0.245 |
| Three-group post hoc | Nodal local efficiency | LOAD vs HC | Cingulum Mid R | -2.151 | 0.033 | 0.245 |
| Three-group post hoc | Nodal local efficiency | LOAD vs HC | ParaHippocampal R | -2.463 | 0.015 | 0.245 |
| Three-group post hoc | Nodal local efficiency | LOAD vs HC | Occipital Sup R | -2.182 | 0.030 | 0.245 |
| Three-group post hoc | Nodal local efficiency | LOAD vs HC | Occipital Inf L | -1.997 | 0.047 | 0.245 |
| Three-group post hoc | Nodal local efficiency | LOAD vs HC | Occipital Inf R | -2.545 | 0.012 | 0.245 |
| Three-group post hoc | Nodal local efficiency | LOAD vs HC | Fusiform L | -2.149 | 0.033 | 0.245 |
| Three-group post hoc | Nodal local efficiency | LOAD vs HC | Angular L | -2.002 | 0.047 | 0.245 |
| Three-group post hoc | Nodal local efficiency | LOAD vs HC | Heschl L | -3.121 | 0.002 | 0.095 |
| Three-group post hoc | Nodal local efficiency | LOAD vs HC | Heschl R | -2.335 | 0.021 | 0.245 |
| Three-group post hoc | Nodal local efficiency | LOAD vs HC | Temporal Sup R | -2.199 | 0.029 | 0.245 |
| Three-group post hoc | Nodal local efficiency | LOAD vs HC | Temporal Mid L | -2.001 | 0.047 | 0.245 |
| Three-group post hoc | Nodal local efficiency | LOAD vs HC | Temporal Pole Mid R | -2.070 | 0.040 | 0.245 |
| Three-group post hoc | Nodal local efficiency | LOAD vs HC | Cerebelum 4 5 L | -2.213 | 0.028 | 0.245 |
| Three-group post hoc | Nodal shortest path length | EOAD vs HC | Calcarine R | 2.439 | 0.016 | 0.917 |
| Three-group post hoc | Nodal shortest path length | EOAD vs HC | Occipital Mid L | 2.571 | 0.011 | 0.917 |

***Footnote.*** *Group coding was group1 = EOAD, group2 = LOAD, and group3 = healthy controls. All group models adjusted for age and sex. Direct two-group analyses are distinguished from the three-group omnibus and three-group post hoc workflows. For pairwise T tests, positive values indicate higher values in the first-named group and negative values indicate higher values in the second-named group according to GRETNA contrast coding; omnibus F statistics have no direction. Raw P values and Benjamini-Hochberg FDR q values are reported to three decimal places. q values were calculated separately across all 116 AAL nodes within each analysis framework, graph metric, and contrast. The table includes all nodes with raw P < 0.05. Only nodal efficiency in Vermis 9 for the overall three-group effect survived FDR correction (F = 8.031, P < 0.001, q = 0.049). Region labels follow the Automated Anatomical Labeling atlas nomenclature.*

**Supplementary Table S4. Secondary clinical associations of structural-functional coupling markers with six clinical scales, jointly corrected across 30 tests.**

| **Clinical scale** | **Imaging marker** | **N** | **β** | **95% CI** | **P value** | **FDR q value** |
| --- | --- | --- | --- | --- | --- | --- |
| FAQ | Mean structural-functional coupling | 115 | 7.577 | [-1.944, 17.099] | 0.118 | 0.929 |
| FAQ | Right middle frontal gyrus SFC | 102 | 0.312 | [-6.778, 7.402] | 0.930 | 0.962 |
| FAQ | Right orbital middle frontal gyrus SFC | 93 | -4.976 | [-15.376, 5.425] | 0.335 | 0.929 |
| FAQ | Right inferior frontal triangular gyrus SFC | 97 | -1.154 | [-9.110, 6.802] | 0.771 | 0.934 |
| FAQ | Left superior medial frontal gyrus SFC | 91 | 3.295 | [-4.370, 10.959] | 0.392 | 0.929 |
| MoCA | Mean structural-functional coupling | 115 | -4.263 | [-10.607, 2.082] | 0.186 | 0.929 |
| MoCA | Right middle frontal gyrus SFC | 102 | -1.299 | [-6.647, 4.050] | 0.627 | 0.934 |
| MoCA | Right orbital middle frontal gyrus SFC | 93 | -0.984 | [-6.953, 4.986] | 0.738 | 0.934 |
| MoCA | Right inferior frontal triangular gyrus SFC | 97 | -2.020 | [-7.941, 3.900] | 0.495 | 0.929 |
| MoCA | Left superior medial frontal gyrus SFC | 91 | -3.441 | [-9.058, 2.175] | 0.224 | 0.929 |
| MMSE | Mean structural-functional coupling | 115 | -1.095 | [-5.889, 3.700] | 0.652 | 0.934 |
| MMSE | Right middle frontal gyrus SFC | 102 | -0.629 | [-4.220, 2.962] | 0.726 | 0.934 |
| MMSE | Right orbital middle frontal gyrus SFC | 93 | -1.698 | [-6.493, 3.098] | 0.474 | 0.929 |
| MMSE | Right inferior frontal triangular gyrus SFC | 97 | 0.182 | [-3.879, 4.243] | 0.928 | 0.962 |
| MMSE | Left superior medial frontal gyrus SFC | 91 | -2.370 | [-6.211, 1.471] | 0.221 | 0.929 |
| GDS | Mean structural-functional coupling | 115 | -0.334 | [-3.621, 2.952] | 0.841 | 0.962 |
| GDS | Right middle frontal gyrus SFC | 102 | 0.361 | [-2.204, 2.927] | 0.778 | 0.934 |
| GDS | Right orbital middle frontal gyrus SFC | 93 | 1.577 | [-1.109, 4.263] | 0.239 | 0.929 |
| GDS | Right inferior frontal triangular gyrus SFC | 97 | -1.160 | [-4.003, 1.683] | 0.415 | 0.929 |
| GDS | Left superior medial frontal gyrus SFC | 91 | 0.419 | [-2.369, 3.207] | 0.764 | 0.934 |
| CDR-SB | Mean structural-functional coupling | 115 | 0.422 | [-2.128, 2.973] | 0.743 | 0.934 |
| CDR-SB | Right middle frontal gyrus SFC | 102 | 0.813 | [-0.815, 2.441] | 0.320 | 0.929 |
| CDR-SB | Right orbital middle frontal gyrus SFC | 93 | -0.057 | [-2.708, 2.594] | 0.965 | 0.965 |
| CDR-SB | Right inferior frontal triangular gyrus SFC | 97 | 0.317 | [-1.500, 2.135] | 0.727 | 0.934 |
| CDR-SB | Left superior medial frontal gyrus SFC | 91 | 0.797 | [-0.969, 2.564] | 0.369 | 0.929 |
| ADAS | Mean structural-functional coupling | 115 | 7.017 | [-5.615, 19.649] | 0.273 | 0.929 |
| ADAS | Right middle frontal gyrus SFC | 102 | -3.269 | [-10.028, 3.489] | 0.335 | 0.929 |
| ADAS | Right orbital middle frontal gyrus SFC | 93 | -4.013 | [-13.974, 5.948] | 0.416 | 0.929 |
| ADAS | Right inferior frontal triangular gyrus SFC | 97 | -2.681 | [-9.786, 4.425] | 0.451 | 0.929 |
| ADAS | Left superior medial frontal gyrus SFC | 91 | -0.557 | [-7.597, 6.483] | 0.874 | 0.962 |

***Footnote.*** *β values are imaging-marker coefficients from clinical association models in the combined EOAD and LOAD sample, adjusted for age, sex, and LOAD-versus-EOAD subgroup. N is the row-specific complete-case sample requiring available clinical outcome, imaging marker, age, and sex data; healthy controls were not included. Values in square brackets are 95% confidence intervals. The secondary family comprised five structural-functional coupling markers tested against six clinical scales, yielding 30 tests. Joint FDR q values were calculated across all 30 raw P values using the Benjamini-Hochberg procedure. Graph-theoretical, ALFF, and fALFF markers were not included. No association reached nominal significance or survived joint FDR correction; the minimum q value was 0.929. Abbreviations: ADAS, Alzheimer’s Disease Assessment Scale; CDR-SB, Clinical Dementia Rating-Sum of Boxes; FAQ, Functional Activities Questionnaire; FDR, false discovery rate; GDS, Geriatric Depression Scale; MMSE, Mini-Mental State Examination; MoCA, Montreal Cognitive Assessment; SFC, structural-functional coupling.*

**Supplementary Table S5. Complete model-specific estimates for sensitivity analyses of the four frontal regional structural-functional coupling findings.**

| **Model** | **N** | **β** | **95% CI** | **P value** | **FDR q value** |
| --- | --- | --- | --- | --- | --- |
| **Panel A. Right middle frontal gyrus** | | | | | |
| Age + sex | 195 | 0.257 | [0.115, 0.399] | <0.001 | 0.032* |
| Age + sex + education | 195 | 0.258 | [0.115, 0.401] | <0.001 | 0.032* |
| Age + sex + education + MMSE | 195 | 0.257 | [0.114, 0.401] | <0.001 | 0.032* |
| Age + sex + education + MoCA | 195 | 0.256 | [0.113, 0.400] | <0.001 | 0.046* |
| Age + sex + education + FAQ | 195 | 0.258 | [0.113, 0.402] | <0.001 | 0.037* |
| Age + sex + education + GDS | 195 | 0.261 | [0.117, 0.404] | <0.001 | 0.030* |
| Age + sex + education + CDR-SB | 195 | 0.260 | [0.117, 0.403] | <0.001 | 0.028* |
| Age + sex + education + ADAS total score | 195 | 0.261 | [0.118, 0.404] | <0.001 | 0.027* |
| Age + sex + education + bilateral hippocampal volume/eTIV | 195 | 0.272 | [0.121, 0.423] | <0.001 | 0.022* |
| Age + sex + education + bilateral hippocampal volume + eTIV | 195 | 0.260 | [0.105, 0.414] | 0.001 | 0.047* |
| Age + age² + sex + education | 195 | 0.267 | [0.110, 0.424] | 0.001 | 0.063 |
| Age + sex + education + APOE ε4 carrier status | 178 | 0.235 | [0.068, 0.402] | 0.006 | 0.184 |
| Age + sex + education + amyloid PET status | 177 | 0.232 | [0.067, 0.397] | 0.007 | 0.151 |
| Age + sex + education + amyloid centiloids | 177 | 0.235 | [0.072, 0.397] | 0.005 | 0.149 |
| AD-only: age + sex + education + disease stage | 102 | 0.286 | [0.083, 0.488] | 0.007 | 0.468 |
| AD-only: overlap weighting using age, sex, and education | 102 | 0.303 | [0.198, 0.408] | <0.001 | <0.001* |
| Age + sex + mean Power FD | 195 | 0.258 | [0.115, 0.400] | <0.001 | 0.031* |
| Amyloid-negative HC-restricted model: age + sex | 166 | 0.286 | [0.132, 0.440] | <0.001 | 0.047* |
| **Panel B. Right orbital middle frontal gyrus** | | | | | |
| Age + sex | 170 | 0.321 | [0.140, 0.501] | <0.001 | 0.032* |
| Age + sex + education | 170 | 0.33 | [0.149, 0.510] | <0.001 | 0.032* |
| Age + sex + education + MMSE | 170 | 0.326 | [0.148, 0.504] | <0.001 | 0.032* |
| Age + sex + education + MoCA | 170 | 0.322 | [0.136, 0.509] | <0.001 | 0.046* |
| Age + sex + education + FAQ | 170 | 0.323 | [0.143, 0.504] | <0.001 | 0.037* |
| Age + sex + education + GDS | 170 | 0.34 | [0.159, 0.520] | <0.001 | 0.030* |
| Age + sex + education + CDR-SB | 170 | 0.325 | [0.141, 0.510] | <0.001 | 0.028* |
| Age + sex + education + ADAS total score | 170 | 0.338 | [0.155, 0.520] | <0.001 | 0.027* |
| Age + sex + education + bilateral hippocampal volume/eTIV | 170 | 0.372 | [0.191, 0.553] | <0.001 | 0.014* |
| Age + sex + education + bilateral hippocampal volume + eTIV | 170 | 0.379 | [0.196, 0.561] | <0.001 | 0.012* |
| Age + age² + sex + education | 170 | 0.373 | [0.175, 0.570] | <0.001 | 0.042* |
| Age + sex + education + APOE ε4 carrier status | 136 | 0.309 | [0.101, 0.516] | 0.0043 | 0.165 |
| Age + sex + education + amyloid PET status | 138 | 0.289 | [0.089, 0.489] | 0.0054 | 0.151 |
| Age + sex + education + amyloid centiloids | 138 | 0.337 | [0.134, 0.540] | 0.0016 | 0.130 |
| AD-only: age + sex + education + disease stage | 93 | 0.333 | [0.065, 0.602] | 0.017 | 0.468 |
| AD-only: overlap weighting using age, sex, and education | 93 | 0.434 | [0.358, 0.510] | <0.001 | <0.001* |
| Age + sex + mean Power FD | 170 | 0.316 | [0.136, 0.496] | <0.001 | 0.031* |
| Amyloid-negative HC-restricted model: age + sex | 158 | 0.32 | [0.119, 0.522] | 0.0024 | 0.094 |
| **Panel C. Right inferior frontal triangular gyrus** | | | | | |
| Age + sex | 177 | 0.242 | [0.102, 0.383] | <0.001 | 0.032* |
| Age + sex + education | 177 | 0.243 | [0.101, 0.384] | <0.001 | 0.033* |
| Age + sex + education + MMSE | 177 | 0.243 | [0.101, 0.384] | 0.001 | 0.032* |
| Age + sex + education + MoCA | 177 | 0.239 | [0.097, 0.381] | 0.001 | 0.046* |
| Age + sex + education + FAQ | 177 | 0.244 | [0.102, 0.387] | <0.001 | 0.037* |
| Age + sex + education + GDS | 177 | 0.238 | [0.095, 0.380] | 0.001 | 0.038* |
| Age + sex + education + CDR-SB | 177 | 0.244 | [0.102, 0.387] | <0.001 | 0.028* |
| Age + sex + education + ADAS total score | 177 | 0.246 | [0.104, 0.389] | <0.001 | 0.033* |
| Age + sex + education + bilateral hippocampal volume/eTIV | 177 | 0.234 | [0.090, 0.377] | 0.002 | 0.0497* |
| Age + sex + education + bilateral hippocampal volume + eTIV | 177 | 0.237 | [0.091, 0.383] | 0.002 | 0.052 |
| Age + age² + sex + education | 177 | 0.187 | [0.032, 0.343] | 0.018 | 0.400 |
| Age + sex + education + APOE ε4 carrier status | 163 | 0.253 | [0.095, 0.411] | 0.002 | 0.119 |
| Age + sex + education + amyloid PET status | 162 | 0.242 | [0.084, 0.400] | 0.003 | 0.151 |
| Age + sex + education + amyloid centiloids | 162 | 0.249 | [0.092, 0.405] | 0.002 | 0.130 |
| AD-only: age + sex + education + disease stage | 97 | 0.275 | [0.071, 0.478] | 0.009 | 0.468 |
| AD-only: overlap weighting using age, sex, and education | 97 | 0.117 | [0.031, 0.203] | 0.009 | 0.019* |
| Age + sex + mean Power FD | 177 | 0.242 | [0.101, 0.383] | <0.001 | 0.031* |
| Amyloid-negative HC-restricted model: age + sex | 163 | 0.257 | [0.105, 0.409] | 0.001 | 0.069 |
| **Panel D. Left superior medial frontal gyrus** | | | | | |
| Age + sex | 176 | 0.219 | [0.090, 0.348] | 0.001 | 0.032* |
| Age + sex + education | 176 | 0.219 | [0.089, 0.348] | 0.001 | 0.033* |
| Age + sex + education + MMSE | 176 | 0.217 | [0.089, 0.345] | 0.001 | 0.032* |
| Age + sex + education + MoCA | 176 | 0.210 | [0.082, 0.339] | 0.002 | 0.046* |
| Age + sex + education + FAQ | 176 | 0.213 | [0.083, 0.344] | 0.002 | 0.047* |
| Age + sex + education + GDS | 176 | 0.218 | [0.088, 0.349] | 0.001 | 0.038* |
| Age + sex + education + CDR-SB | 176 | 0.224 | [0.094, 0.354] | <0.001 | 0.028* |
| Age + sex + education + ADAS total score | 176 | 0.221 | [0.090, 0.352] | 0.001 | 0.033* |
| Age + sex + education + bilateral hippocampal volume/eTIV | 176 | 0.254 | [0.122, 0.387] | <0.001 | 0.014* |
| Age + sex + education + bilateral hippocampal volume + eTIV | 176 | 0.250 | [0.115, 0.384] | <0.001 | 0.022* |
| Age + age² + sex + education | 176 | 0.183 | [0.040, 0.326] | 0.013 | 0.400 |
| Age + sex + education + APOE ε4 carrier status | 167 | 0.237 | [0.089, 0.385] | 0.002 | 0.119 |
| Age + sex + education + amyloid PET status | 167 | 0.197 | [0.050, 0.344] | 0.009 | 0.152 |
| Age + sex + education + amyloid centiloids | 167 | 0.201 | [0.055, 0.347] | 0.008 | 0.149 |
| AD-only: age + sex + education + disease stage | 91 | 0.222 | [0.035, 0.409] | 0.021 | 0.468 |
| AD-only: overlap weighting using age, sex, and education | 91 | 0.152 | [0.073, 0.232] | <0.001 | 0.001* |
| Age + sex + mean Power FD | 176 | 0.220 | [0.090, 0.349] | 0.001 | 0.031* |
| Amyloid-negative HC-restricted model: age + sex | 158 | 0.195 | [0.056, 0.335] | 0.007 | 0.157 |

Footnote. β values represent the adjusted LOAD-versus-EOAD effect; positive values indicate higher regional structural-functional coupling in LOAD. N is the total model- and ROI-specific complete-case sample for models including healthy controls and is the EOAD-plus-LOAD complete-case sample for models labeled “AD-only.” The amyloid-negative-control rows report the total ROI-specific sample from the corresponding restricted three-group models and therefore match Supplementary Table S6. Values in square brackets are 95% confidence intervals. Benjamini-Hochberg q values were corrected across 116 AAL regions within each model, and * indicates q < 0.05. Clinical and severity covariates were entered separately. The overlap-weighted models used disease-only weights estimated from age, sex, and education. The amyloid-negative-control models retained disease participants and restricted healthy controls to participants with negative amyloid PET status. “AD-only” denotes analyses restricted to the diagnosis-age-defined EOAD and LOAD disease groups, which included MCI and dementia participants. Abbreviations: AAL, Automated Anatomical Labeling; ADAS, Alzheimer’s Disease Assessment Scale; APOE, apolipoprotein E; CDR-SB, Clinical Dementia Rating-Sum of Boxes; eTIV, estimated total intracranial volume; FAQ, Functional Activities Questionnaire; FD, framewise displacement; FDR, false discovery rate; GDS, Geriatric Depression Scale; HC, healthy controls; MMSE, Mini-Mental State Examination; MoCA, Montreal Cognitive Assessment; PET, positron emission tomography.

**Supplementary Table S6. Structural-functional coupling sensitivity analysis with healthy controls restricted to amyloid-negative participants.**

**Panel A. Sample counts after restriction**

| **Group** | **Original multimodal N** | **Restricted N** |
| --- | --- | --- |
| EOAD | 47 | 47 |
| LOAD | 69 | 69 |
| HC | 111 | 68 |

**Panel B. Global mean SFC contrasts in the restricted three-group sample**

| **Contrast** | **N** | **β** | **95% CI** | **P value** | **FDR q value** |
| --- | --- | --- | --- | --- | --- |
| LOAD vs EOAD | 184 | 0.029 | [-0.019, 0.077] | 0.239 | 0.468 |
| Amyloid-negative HC vs EOAD | 184 | 0.015 | [-0.026, 0.057] | 0.468 | 0.468 |
| Amyloid-negative HC vs LOAD | 184 | -0.014 | [-0.048, 0.021] | 0.441 | 0.468 |

**Panel C. Frontal ROI-level SFC contrasts in the restricted three-group sample**

| **ROI** | **Contrast** | **N** | **β** | **95% CI** | **P value** | **FDR q value** |
| --- | --- | --- | --- | --- | --- | --- |
| Right middle frontal gyrus | LOAD vs EOAD | 166 | 0.286 | [0.132, 0.440] | <0.001 | 0.047 |
| Right middle frontal gyrus | Amyloid-negative HC vs EOAD | 166 | 0.211 | [0.073, 0.348] | 0.003 | 0.365 |
| Right middle frontal gyrus | Amyloid-negative HC vs LOAD | 166 | -0.075 | [-0.182, 0.031] | 0.163 | 0.735 |
| Right orbital middle frontal gyrus | LOAD vs EOAD | 158 | 0.320 | [0.119, 0.522] | 0.002 | 0.094 |
| Right orbital middle frontal gyrus | Amyloid-negative HC vs EOAD | 158 | 0.142 | [-0.043, 0.328] | 0.130 | 0.829 |
| Right orbital middle frontal gyrus | Amyloid-negative HC vs LOAD | 158 | -0.178 | [-0.305, -0.050] | 0.007 | 0.212 |
| Right inferior frontal triangular gyrus | LOAD vs EOAD | 163 | 0.257 | [0.105, 0.409] | 0.001 | 0.069 |
| Right inferior frontal triangular gyrus | Amyloid-negative HC vs EOAD | 163 | 0.137 | [0.001, 0.273] | 0.049 | 0.758 |
| Right inferior frontal triangular gyrus | Amyloid-negative HC vs LOAD | 163 | -0.121 | [-0.225, -0.017] | 0.024 | 0.379 |
| Left superior medial frontal gyrus | LOAD vs EOAD | 158 | 0.195 | [0.056, 0.335] | 0.007 | 0.157 |
| Left superior medial frontal gyrus | Amyloid-negative HC vs EOAD | 158 | 0.053 | [-0.071, 0.176] | 0.397 | 0.909 |
| Left superior medial frontal gyrus | Amyloid-negative HC vs LOAD | 158 | -0.143 | [-0.239, -0.047] | 0.004 | 0.197 |

***Footnote.*** *The control group was restricted to participants with negative amyloid PET status, reducing healthy controls from 111 to 68; all EOAD and LOAD participants were retained. Models adjusted for age and sex. Panel B contrasts were estimated from one three-group model; N is the total complete-case sample across EOAD, LOAD, and amyloid-negative healthy controls and is therefore shared by all three contrasts. For each ROI in Panel C, the three contrasts were estimated from the same ROI-specific three-group model; N is the total ROI-specific complete-case sample across the three groups and is shared across contrasts within that ROI. ROI-level q values were corrected across 116 AAL regions separately for each contrast.*

**Supplementary Table S7. Seed-based resting-state functional connectivity clusters surviving voxel-level FDR correction.**

| **Contrast** | **Region** | **Peak coordinate (x, y, z); k** | **Peak T** | **FDR q** |
| --- | --- | --- | --- | --- |
| EOAD > HC | Cerebellum Crus2 L | (-39, -60, -42); k = 54 | 5.38 | 0.004 |
| EOAD > HC | Lingual L | (-27, -78, -15); k = 97 | 5.17 | 0.010 |
| EOAD > HC | Cerebellum Crus1 R | (18, -75, -24); k = 78 | 5.03 | 0.010 |
| EOAD > HC | Cerebellum Crus2 L | (-42, -54, -39); k = 89 | 4.97 | 0.010 |
| EOAD > HC | Cerebellum 6 L | (-27, -51, -21); k = 18 | 4.60 | 0.020 |
| EOAD > HC | Cerebellum Crus1 L | (-21, -90, -24); k = 11 | 4.43 | 0.023 |
| EOAD > HC | Calcarine R | (15, -60, 9); k = 18 | 4.37 | 0.024 |
| EOAD > HC | Occipital Inf L | (-54, -63, -12); k = 27 | 4.35 | 0.024 |
| EOAD > HC | Cerebellum 8 R | (27, -66, -48); k = 12 | 4.13 | 0.029 |
| EOAD > HC | Calcarine R | (12, -75, 3); k = 17 | 4.03 | 0.029 |
| EOAD > HC | Occipital Mid R | (33, -78, 0); k = 12 | 4.03 | 0.029 |
| EOAD > HC | Occipital Mid L | (-45, -69, 3); k = 13 | 4.01 | 0.029 |
| EOAD > HC | Occipital Inf L | (-39, -72, -9); k = 10 | 4.00 | 0.029 |
| EOAD > HC | Occipital Inf R | (45, -75, -12); k = 19 | 4.00 | 0.029 |
| EOAD > HC | Occipital Inf L | (-30, -87, -3); k = 16 | 3.98 | 0.029 |
| EOAD > HC | Temporal Inf R | (39, -63, -6); k = 39 | 3.95 | 0.029 |
| EOAD > HC | Fusiform L | (-45, -54, -18); k = 14 | 3.95 | 0.029 |
| EOAD > HC | Precuneus L | (-15, -48, 60); k = 19 | 3.90 | 0.029 |
| EOAD > HC | Lingual L | (-24, -60, -3); k = 23 | 3.82 | 0.029 |
| EOAD > HC | Frontal Sup Medial L | (-3, 51, 30); k = 12 | 4.57 | 0.030 |
| EOAD > HC | Temporal Mid L | (-57, -39, 6); k = 20 | 4.48 | 0.030 |
| EOAD > HC | Cerebellum Crus1 R | (18, -75, -30); k = 24 | 4.38 | 0.030 |
| EOAD > HC | Occipital Mid L | (-45, -69, 3); k = 22 | 4.21 | 0.030 |
| EOAD > HC | Cingulum Mid L | (-3, -39, 48); k = 19 | 4.17 | 0.030 |
| EOAD > HC | Fusiform R | (24, -60, -12); k = 12 | 4.13 | 0.030 |
| EOAD > HC | Temporal Inf R | (39, -63, -6); k = 25 | 4.13 | 0.030 |
| EOAD > HC | Cerebellum Crus1 L | (-9, -69, -24); k = 16 | 4.12 | 0.030 |
| EOAD > HC | Temporal Mid L | (-54, -69, 18); k = 15 | 4.11 | 0.030 |
| EOAD > HC | Cerebellum Crus1 L | (-39, -60, -27); k = 15 | 4.10 | 0.030 |
| EOAD > HC | Temporal Inf L | (-54, -60, -12); k = 13 | 4.04 | 0.030 |
| EOAD > HC | Temporal Mid R | (42, -60, 3); k = 11 | 3.74 | 0.033 |
| EOAD > HC | Occipital Inf L | (-45, -66, -12); k = 10 | 3.81 | 0.033 |
| EOAD > HC | Cerebellum 9 L | (-18, -48, -51); k = 10 | 3.61 | 0.034 |
| EOAD > HC | Precuneus L | (-6, -60, 39); k = 10 | 3.60 | 0.034 |
| LOAD > HC | Putamen R | (21, 24, -3); k = 11 | 5.69 | 0.001 |
| LOAD > HC | Precuneus R | (9, -60, 63); k = 37 | 4.83 | 0.012 |
| LOAD > HC | Parietal Inf L | (-27, -45, 48); k = 21 | 4.65 | 0.016 |
| LOAD > HC | Occipital Mid R | (27, -72, 33); k = 17 | 4.53 | 0.017 |
| LOAD > HC | Cingulum Mid L | (-3, -15, 42); k = 12 | 4.52 | 0.017 |
| LOAD > HC | Precuneus L | (-3, -48, 54); k = 22 | 4.41 | 0.017 |
| LOAD > HC | Parietal Sup L | (-24, -48, 66); k = 10 | 4.38 | 0.017 |
| LOAD > HC | Cingulum Mid R | (12, -33, 39); k = 10 | 4.21 | 0.020 |
| LOAD > HC | Cuneus R | (18, -63, 39); k = 11 | 3.89 | 0.026 |

***Footnote.*** *Peak coordinate denotes the Montreal Neurological Institute coordinate of the peak voxel, and k denotes cluster extent in voxels. Entries are grouped by contrast and ordered by FDR q value. FDR q values were corrected at the voxel level within each contrast. No EOAD-versus-LOAD cluster survived voxel-level FDR correction.*

**Supplementary Table S8. Representative exploratory regional ALFF and fALFF findings.**

| **Feature** | **Contrast** | **ROI / Region** | **Result** |
| --- | --- | --- | --- |
| fALFF | LOAD vs EOAD | Temporal Mid L | β = 0.138; p < 0.001 |
| fALFF | LOAD vs EOAD | Temporal Pole Sup R | β = 0.152; p = 0.013 |
| fALFF | LOAD vs EOAD | Occipital Inf R | β = -0.174; p = 0.013 |
| fALFF | LOAD vs EOAD | Cerebellum Crus I L | β = -0.0949; p = 0.057 |
| ALFF | LOAD vs EOAD | Pallidum L | β = -0.111; p = 0.131 |
| ALFF | LOAD vs EOAD | Caudate R | β = 0.0726; p = 0.305 |

***Footnote.*** *Representative ALFF and fALFF findings are shown to document the exploratory local-functional search space. P values are uncorrected and are presented descriptively. These findings are not interpreted as primary subtype-discriminative evidence. β values are adjusted LOAD-versus-EOAD estimates; positive values indicate higher signal in LOAD.*
